# A novel immune classification reveals distinct immune escape mechanism and genomic alterations: implications for immunotherapy in hepatocellular carcinoma

**DOI:** 10.1186/s12967-020-02697-y

**Published:** 2021-01-06

**Authors:** Zaoqu Liu, Yuyuan Zhang, Chengcheng Shi, Xueliang Zhou, Kaihao Xu, Dechao Jiao, Zhenqiang Sun, Xinwei Han

**Affiliations:** 1 Department of Interventional Radiology, The First Affiliated Hospital of Zhengzhou University, Zhengzhou, 450052 Henan China; 2 Department of Colorectal Surgery, The First Affiliated Hospital of Zhengzhou University, Zhengzhou, 450052 Henan China

**Keywords:** Hepatocellular carcinoma, Immunotherapy, Tumor immunological microenvironment, Immune escape, Molecular subtype

## Abstract

**Background:**

The tumor immunological microenvironment (TIME) has a prominent impact on prognosis and immunotherapy. However, the heterogeneous TIME and the mechanisms by which TIME affects immunotherapy have not been elucidated in hepatocellular carcinoma (HCC).

**Methods:**

A total of 2195 eligible HCC patients from TCGA and GEO database were collected. We comprehensively explored the different heterogeneous TIME phenotypes and its clinical significance. The potential immune escape mechanisms and what genomic alterations may drive the formation of different phenotypes were further investigated.

**Results:**

We identified three phenotypes in HCC: TIME-1, the “immune-deficiency” phenotype, with immune cell depletion and proliferation; TIME-2, the “immune-suppressed” phenotype, with enrichment of immunosuppressive cells; TIME-3, the “immune-activated phenotype”, with abundant leukocytes infiltration and immune activation. The prognosis and sensitivity to both sorafenib and immunotherapy differed among the three phenotypes. We also underlined the potential immune escape mechanisms: lack of leukocytes and defective tumor antigen presentation capacity in TIME-1, increased immunosuppressive cells in TIME-2, and rich in immunoinhibitory molecules in TIME-3. The different phenotypes also demonstrated specific genomic events: TIME-1 characterized by TP53, CDKN2A, CTNNB1, AXIN1 and FOXD4 alterations; TIME-2 characterized by significant alteration patterns in the PI3K pathway; TIME-3 characterized by ARID1A mutation. Besides, the TIME index (TI) was proposed to quantify TIME infiltration pattern, and it was a superior prognostic and immunotherapy predictor. A pipeline was developed to classify single patient into one of these three subtypes and calculated the TI.

**Conclusions:**

We identified three TIME phenotypes with different clinical outcomes, immune escape mechanisms and genomic alterations in HCC, which could present strategies for improving the efficacy of immunotherapy. TI as a novel prognostic and immunotherapeutic signature that could guide personalized immunotherapy and clinical management of HCC.

## Background

Hepatocellular carcinoma (HCC) is the dominant histologic type of primary liver cancer, with a high incidence and mortality rate [1]. Although there are various therapeutic modalities for HCC, including surgical resection, chemotherapy, radiofrequency ablation and liver transplantation, its recurrence rate and prognosis remains unsatisfactory [2, 3]. Recently, immunotherapy has made great progress as a new treatment method in HCC. However, to date, this only benefited a subset of patients [4, 5]. The insufficient understanding of the tumor immunological microenvironment (TIME) may be the main reason for disappointing results. At the individual level, HCC has significant TIME heterogeneity, and the comprehensive understanding of the heterogeneity was crucial for clinical diagnosis, personalized treatment and prognosis prediction in HCC [6].

HCC is a typical inflammation-driven tumor, which is mainly derived from viral infections and liver fibrosis [7]. The transition from chronic hepatitis to HCC is accompanied by changes in local TIME [8]. Immune cells are the main components of TIME, and their number and status play a critical role in the progression of tumor development, invasion and metastasis. Previous researches have mainly focused on one or several immune cell types [9–12], which may bias the understanding of TIME due to the intensive cellular interaction between different cells. Hence, it is essential to be considered as a whole.

The rapid development of genomics and transcriptomics has made it possible to systematically explore the TIME heterogeneity in HCC. In the present study, we collected a total of 2,175 eligible samples from 15 cohorts, and combined with multi-omics data, hoping to explore different heterogeneous TIME phenotypes, further investigate the potential immune escape mechanisms of each TIME phenotype and what genomic alterations may lead to the formation of these different phenotypes. As a result, we successfully identified and validated three heterogeneous phenotypes based on the broad-spectrum immune cells in TIME. These three phenotypes exhibited different clinical outcomes, immune escape mechanisms and specific genomic alterations. In addition, the TIME index (TI) was developed to quantify TIME infiltration pattern, and it was a superior prognostic and immunotherapy predictor.

## Methods

### Data collecting and processing

The present workflow was shown in Additional file 1: Fig. S1. For the discovery cohorts, the HCC microarray datasets were recruited from the Gene Expression Ominibus (GEO) database with the following criteria: (1) only from Affymetrix platform; (2) primary liver cancer; (3) untreated patients; (4) the number of patients was ≥ 50; (5) with more than 12,000 protein coding genes. Finally, 14 eligible datasets containing 1,821 patients were retrieved (Additional file 2: Table S1). For the TCGA validation cohort, the TCGA-LIHC RNA-seq data was obtained from the UCSC Xena Portal. Please refer to Additional file 3: Materials and Methods for the data processing details. The corresponding clinical and sample information were obtained from the GEO and UCSC databases. For the TCGA-LIHC project, the somatic mutation data, copy number variation data, and DNA methylation data were obtained from the TCGA portal. In addition, we also downloaded the RNA-seq data and clinical information of the 32 other cancer types from the UCSC databases.

### Integrated assessment of the TIME immune cell composition

In order to quantify the relative abundance of each immune cell population in TIME, we applied the single sample gene set enrichment analysis (ssGSEA) algorithm [13]. The gene sets for marking 24 immune cell types was recruited from Bindea et al. study [14]. In order to ensure the rationality and robustness of the ssGSEA results, two different algorithms were utilized to further validate: CIBERSORT [15] and MCP-counter [16]. The details were described in Additional file 3: Materials and Methods.

### Identification and validation of the TIME phenotypes

We used the ConsensusClusterPlus package to determine the optimum number of clusters in the GEO cohort [17]. The results were further detected using the cumulative distribution function (CDF) curve, proportion of ambiguous clustering (PAC) score, and Nbclust [18]. To evaluate the reproducibility of the clusters generated from consensus clustering in the GEO cohort, the in-group proportion (IGP) statistical analysis was employed to further validate the existence of these clusters in the TCGA validation cohort [19]. The details were described in Additional file 3: Materials and Methods.

### Gene set variation analysis (GSVA)

To further explore the potential biological function and progress variations of each phenotype, we conducted the GSVA analysis via GSVA package [20]. The gene sets, including the Hallmark and KEGG gene sets, were derived from the Molecular Signatures Database (MSigDB). The limma package was implemented to identify the significantly altered pathways between each phenotype and the others with the following threshold: log FC > 0.2 and adjusted P-value < 0.05. The resulting P-values from Benjamini-Hochberg (BH) multi-test correction were adjusted for multiple comparisons using the false discovery rate (FDR).

### Assessment of immunotherapy and sorafenib

The Tumor Immune Dysfunction and Exclusion (TIDE) web application (http://tide.dfci.harvard.edu) was employed to predict the immunotherapy response of each patient [21]. TIDE algorithm was a computational method to model two primary mechanisms of tumor immune evasion: the induction of T cell dysfunction in tumors with high infiltration of cytotoxic T lymphocytes (CTL) and the prevention of T cell infiltration in tumors with low CTL level. The Subclass Mapping (SubMap) method was utilized to evaluate the expression similarity between the three phenotypes and the patients with different immunotherapy responses [22]. The SubMap employ GSEA algorithm to deduce the extent of commonality of the two groups. An adjust P-value < 0.05 suggest the significant similarity between two groups. We further applied pRRophetic package to estimate the chemotherapeutic response of sorafenib, which fitted the ridge regression model based on baseline gene expression and drug sensitivity of the cell line, thus allowing the prediction of the clinical chemotherapeutic response using only patients’ baseline gene expression data.

### Analysis of immunogenomic features

We calculated or collected tumor mutation burden (TMB), SNV or Indel neoantigen load, aneuploidy scores (AS), homologous recombination defects (HRD) score, microsatellite instability (MSI), TCR or BCR diversity, cancer/testis-antigens (CTAs) level, antigen processing and presenting machinery scores (APS), and MHC-related molecules, in order to investigate the tumor immunogenicity of HCC. The details were described in Additional file 3: Materials and Methods.

### Multi-omics profiling of immunomodulators

A total of 62 immunomodulators (including 12 MHC class I genes, 11 MHC class II genes, 27 checkpoint stimulator genes, and 12 checkpoint inhibitor genes) were recruited [23]. We investigated the multi-omics regulation landscape (including genes expression, somatic mutation, copy number variation (CNV), DNA methylation and miRNA expression) of 62 immunomodulators in three phenotypes. The Kruskal–Wallis test was performed for the mRNA expression, and Fisher’s test was performed for the somatic mutation and CNV of immunomodulators. The adjusted P-values were acquired using the BH multi-test correction. To survey the correlation between DNA methylation and gene expression of immunomodulators, each methylation site was matched to the corresponding gene. Most of the single genes had multiple methylation sites. In each phenotype, we assessed the Spearman’s correlation between each immunomodulator expression and all the corresponding methylation sites. Subsequently, we obtained a single correlation value for every gene by averaging the corresponding correlation coefficients. Next, we estimated the pattern that the miRNA modulated the immunomodulator expression. The significant inversely correlative pairs of miRNA and immunomodulator were included (Spearman correlation ≤ -0.2 and BH-corrected P < 0.05) within each phenotype. Then, according to the predicted blinding targets for miRNA, these were curated from the miRDB database.

### Driver mutation genes and mutation signatures

We utilized MutSigCV (version 1.41) to identify the significantly mutated genes (SMGs) for three phenotypes of TCGA-LIHC cohort [24], and a q-value of < 0.05 was considered as the threshold. Subsequently, the MutationalPatterns R package was applied to extract the mutation signatures of each phenotype [25]. The mutational signatures can be extracted from mutation count matrix using non-negative matrix factorization (NMF). The optimal factorization rank, which was the number of mutational signatures, can be determined using the NMF package. After calculating the pairwise cosine similarity between the extracted mutation signatures and the 30 COSMIC signatures previously reported (http://cancer.sanger.ac.uk/cosmic/signatures), these extracted mutation signatures were then named based on the COSMIC signature.

### Copy number variations

The TCGAbiolinks R package was used to download the CNV data based on the segment mean value (log2(copy-number/2)) obtained from TCGA database. The ABSOLUTE algorithm was implemented to estimate tumor ploidy for each sample [26]. To quantify the overall fraction of genomic alteration in three phenotypes, we calculated the fraction of genome alteration (FGA), fraction of genome gained (FGG), and fraction of genome lost (FGL). The FGA for a sample was defined as the ratio of the number of bases with CNVs to the number of all bases. The FGG or FGL considered only CNVs that were gained or lost. The GISTIC 2.0 was applied to define the recurrently amplified and deleted regions of each phenotype [27].

### Methylation profiling

We downloaded the HumanMethylation450 array for HCC in TCGA. The global methylation level (GML) was estimated through averaged beta values of the specific probes, as described by Jung et al. [28]. The CD8+ T cell infiltrate status and proliferation score were both derived from Thorsson et al. study [23]. For each phenotype, we identified the epigenetically silenced genes (ESGs) using the following criteria: (1) excluding the CpG sites methylated in normal tissues (mean β-value of > 0.2) or less than 10% of the tumor samples; (2) the DNA methylation data was divided into the methylation group and unmethylation group, according to the cutoff (β-value = 0.3); (3) for each probe, if the difference between the corresponding gene mean expression in the unmethylated group and that in the methylated group was > 1.64 standard deviations of the unmethylated group, the probe would be labeled as epigenetically silenced; (4) when multiple probes were assigned to the same gene, the gene with more than half of the corresponding probes were labeled as epigenetically silenced, and identified as ESG.

### TIME index

We applied the limma package to identify the differentially expressed genes (DEGs) between each phenotype and the others using the following thresholds: |log FC|> 1.5 and adjusted P-value < 0.05. Based on these DEGs, ssGSEA was performed to obtain the TIME index (TI) for each patient. Then, we assessed the prognostic value of TI in both the HCC and pancancer cohort. The performance of TI in predicting the response to immunotherapy was further evaluated in six pre-treatment melanoma cohorts with the gene expression profile and immunotherapy information (Additional file 3: Materials and Methods). The immunotherapy response prediction accuracy of the TI was compared with 11 other known biomarkers (CD274, PDCD1, CTLA4, CD8, TMB, T cell clonality, B cell clonality, TIDE, MSI Score, cytolytic activity (CYT)and APS; the details were described in Additional file 3: Materials and Methods). The area under the ROC curve was used as the quality metric of prediction. In addition, based on the nearest centroid method and Pearson’s correlation, we developed a pipeline to classify single patient into one of these three subtypes and calculated the TI (https://github.com/Zaoqu-Liu/TIME).

## Results

### Immune cell infiltration patterns of TIME

**Fig. 1:**
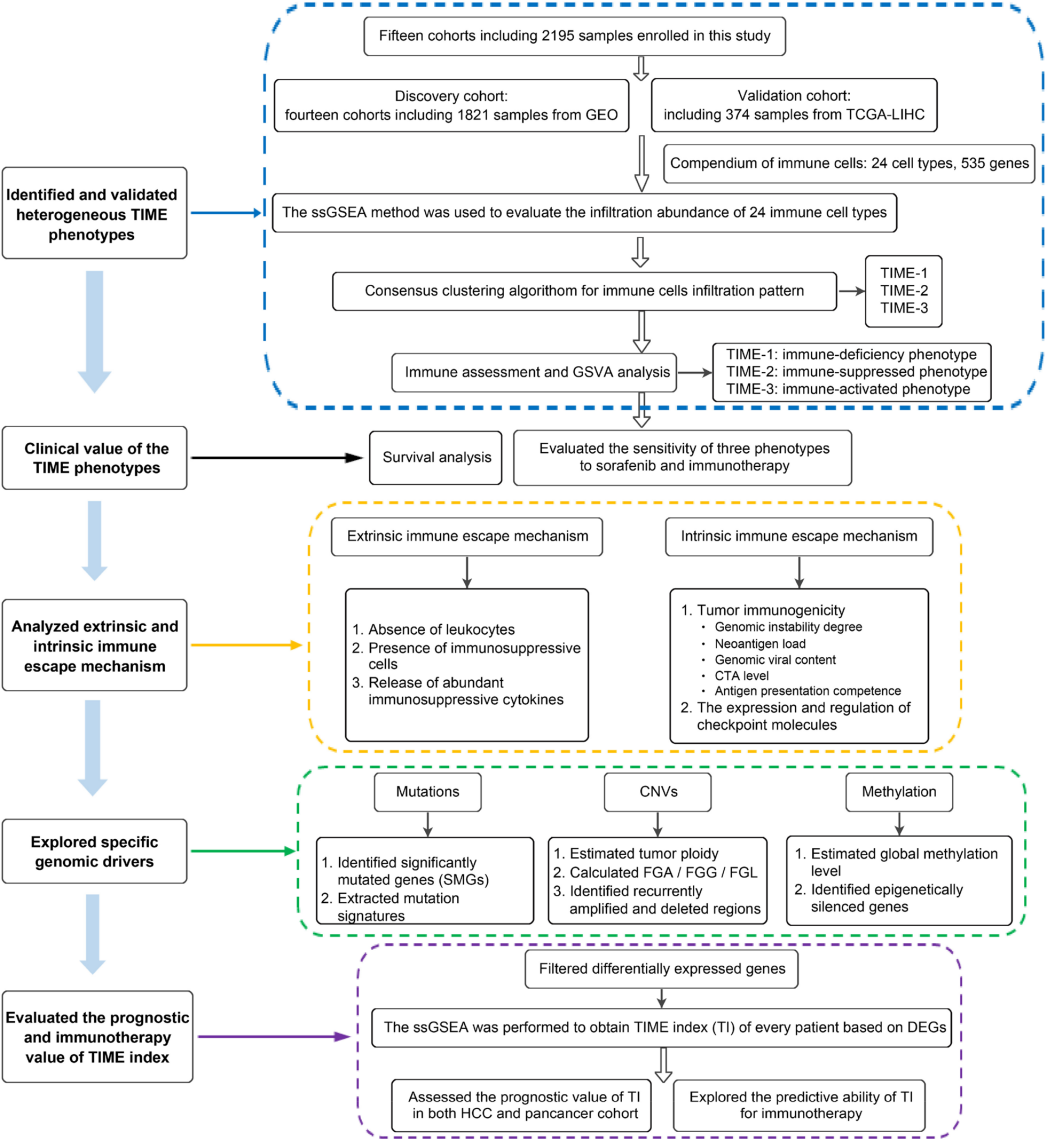
Workflow of our research

**Fig. 2:**
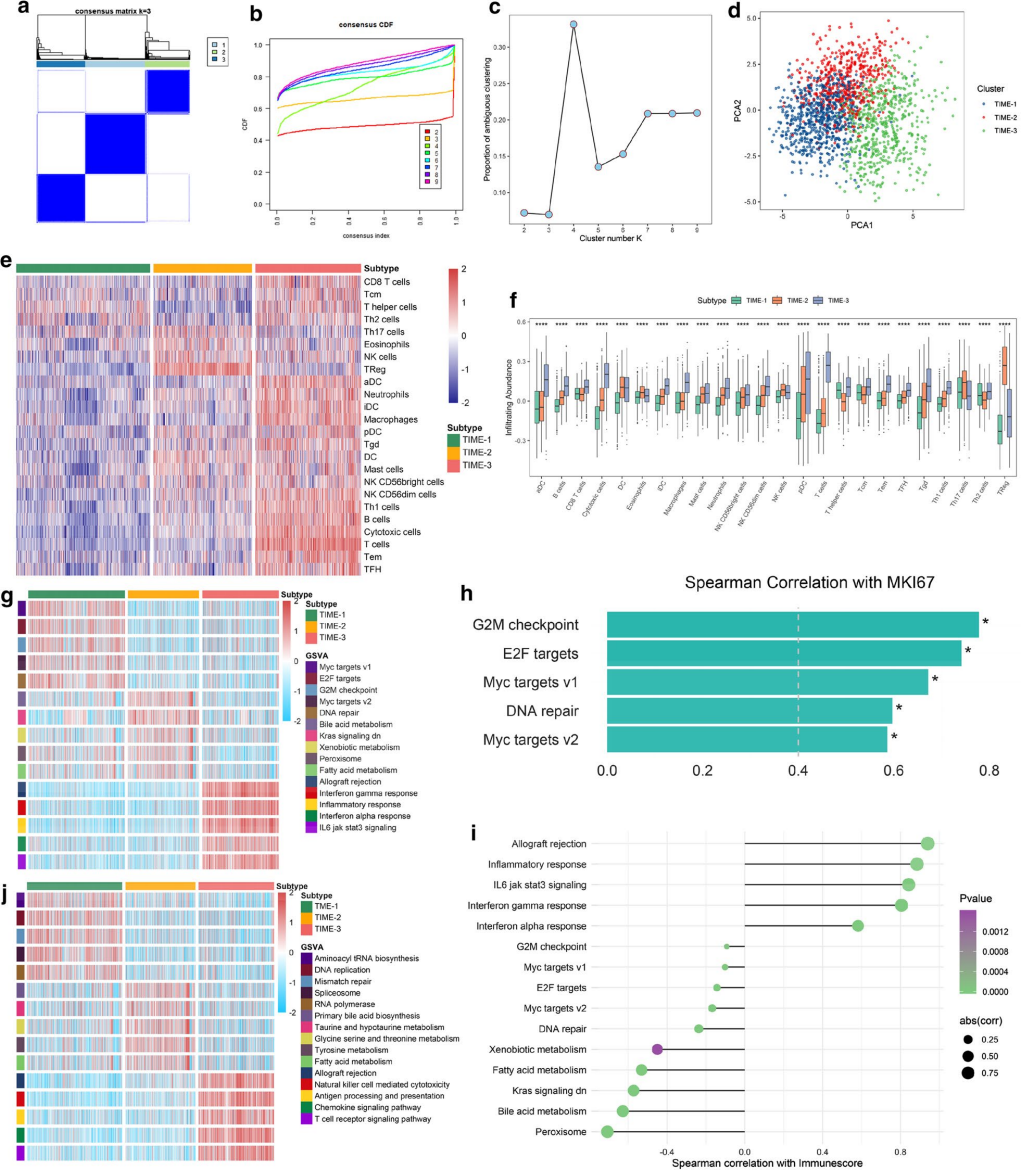
The immune cells infiltration and biological function landscape of the TIME phenotypes. **a** The consensus score matrix of all samples when k = 3. A higher consensus score between two samples indicates they are more likely to be grouped into the same cluster in different iterations. **b** The cumulative distribution functions of consensus matrix for each k (indicated by colors). **c** The proportion of ambiguous clustering (PAC) score, a low value of PAC implies a flat middle segment, allowing conjecture of the optimal k (k = 3) by the lowest PAC. **d** two-dimensional principle component plot by infiltration profile of 24 immune cell subsets. Each point represents a single sample, with different colors indicating the TIME phenotypes. **e** The infiltration abundance of 24 immune cell subsets evaluated by ssGSEA algorithm for three TIME phenotypes in the GEO cohort. **f** The differences of 24 immune cell subsets infiltration among three TIME phenotypes in the GEO cohort. **g** The activation states of Hallmark pathways of distinct TIME phenotypes in the GEO cohort. **h** Spearman correlation of specific Hallmark pathways in TIME-2 with MKI67 (*P < 0.05). **i** Spearman correlation between specific Hallmark pathways in three TIME phenotypes and immune score assessed by ESTIMATE algorithm. **j** The activation states of KEGG pathways of distinct TIME phenotypes in the GEO cohort. For the boxplot, the asterisks represented the statistical p value (*P < 0.05, **P < 0.01, *** P < 0.001, **** P < 0.0001)

We applied the ssGSEA method to assess the infiltration abundance of 24 immune cell types for 1821 HCC samples. The correlation between these immune cells was presented in Additional file 1: Fig. S1A. It was observed that several pairs strongly correlated with immune cells, such as T cell-cytotoxic cells, B cell-T cells and macrophage-immature DC cells. Subsequently, we performed a consensus cluster analysis, in which all HCC samples were initially grouped into different k (k = 2–9) clusters. The CDF curves of the consensus score and PAC value suggested that the optimal division was achieved when k = 3 (Fig. 2a–c). The same result was obtained from NbClust (Additional file 1: Fig. S1B). The three clusters of samples were separated from each other on the two-dimensional principle component plot (Fig. 1d). Thus, based on the infiltration profiles of 24 immune cells in TIME, 1,821 HCC samples were finally classified into three TIME phenotypes (TIME-1 = 721, TIME-2 = 530, TIME-3 = 570). As shown in Fig. 1e, f, TIME-1 presented as an immune deficiency phenotype due to the lowest infiltration in almost all immune cells. On the contrary, it was found that TIME-3 had a significantly higher infiltration level in the majority of immune cells, especially adaptive immune cells (e.g. CD8+ T cells and B cells), suggesting that TIME-3 was associated with immune activation and superior cytotoxic potential. TIME-2 was in an intermediate status of immune infiltration between TIME-1 and TIME-3, and was characterized by higher infiltration immunosuppressive cells that contain Treg and TH17. In addition, it was also observed that there was a richer infiltration in some innate immune cells that contain DC and NK cells in TIME-2. Two other different algorithms were further applied: CIBERSORT and MCP-counter. The results shared a consistent immune infiltration pattern with the ssGSEA method in HCC (Additional file 1: Fig. S1C, D).

In order to ensure the reproducibility and robustness of the TIME phenotypes derived from the GEO cohort, we further conducted the IGP statistical method to validate the TIME phenotypes in the TCGA cohort. The three phenotypes were highly consistent between the discovery and validation cohorts, with the corresponding IGP values at 95.6%, 93.3% and 94.7%, respectively, and the three phenotypes were deemed to be of high-quality due to the statistically significance (all, P < 0.001). The immune cells infiltration patterns in the TCGA cohort exhibited a very similar pattern of immune infiltration to the GEO cohort (Additional file 1: Fig. S1E, F). Furthermore, the NbClust also indicated that the three clusters configuration was “optimal” in the TCGA cohort (Additional file 1: Fig. S1G).

### Specific functional pathways of each TIME phenotype

We further explored the specific functional status and biological mechanisms of each phenotype in the GEO cohort (Fig. 2g and Additional file 2: Table S2). TIME-1 was prominently enriched in pathways, such as Myc targets, G2M checkpoint, and DNA repair. These pathways were remarkably associated with MKI67, and thereby with proliferation (Fig. 2h). Furthermore, it was observed that the immune-relevant pathways were significantly downregulated in TIME-1 (Additional file 2: Table S2). Combined with the lack of immune cell infiltration, we inferred that TIME-1 may present an immune deficiency phenotype. On the contrary, TIME-3 enriched intensive pathways related to immune activation, and these pathways had a remarkably positive association with the immune score assessed through the ESTIMATE algorithm [29] (Fig. 2i), suggesting that TIME-3 may exhibit a state of immune activation. Notably, TIME-2 was significantly upregulated in metabolic-relevant pathways. It was observed that there was significantly negative correlation between the immune score and these specifically activated pathways in TIME-2 (Fig. 2i). Moreover, TIME-2 was rich in immunosuppressive cells (e.g., TH17 cell and Treg), which is known from the previous descriptions. Hence, it was concluded that TIME-2 may present as an immune-suppressed phenotype. The KEGG results was in accordance with the above (Fig. 2j and Additional file 2: Table S3), and similar results were achieved in the TCGA cohort (Additional file 4: Fig. S2A, B and Additional file 2: Table S4, S5). Overall, we identified three TIME phenotypes in HCC showing significantly different immune cell infiltration and biological functions, respectively. TIME-1 was categorized as an immune-deficiency phenotype, characterized by immune cell depletion and proliferation; TIME-2 was categorized as an immune-suppressed phenotype, characterized by enrichment of immunosuppressive cells; TIME-3 was categorized as an immune-activated phenotype, characterized by abundant leukocytes infiltration and immune activation.

### The clinical value of TIME phenotypes

**Fig. 3:**
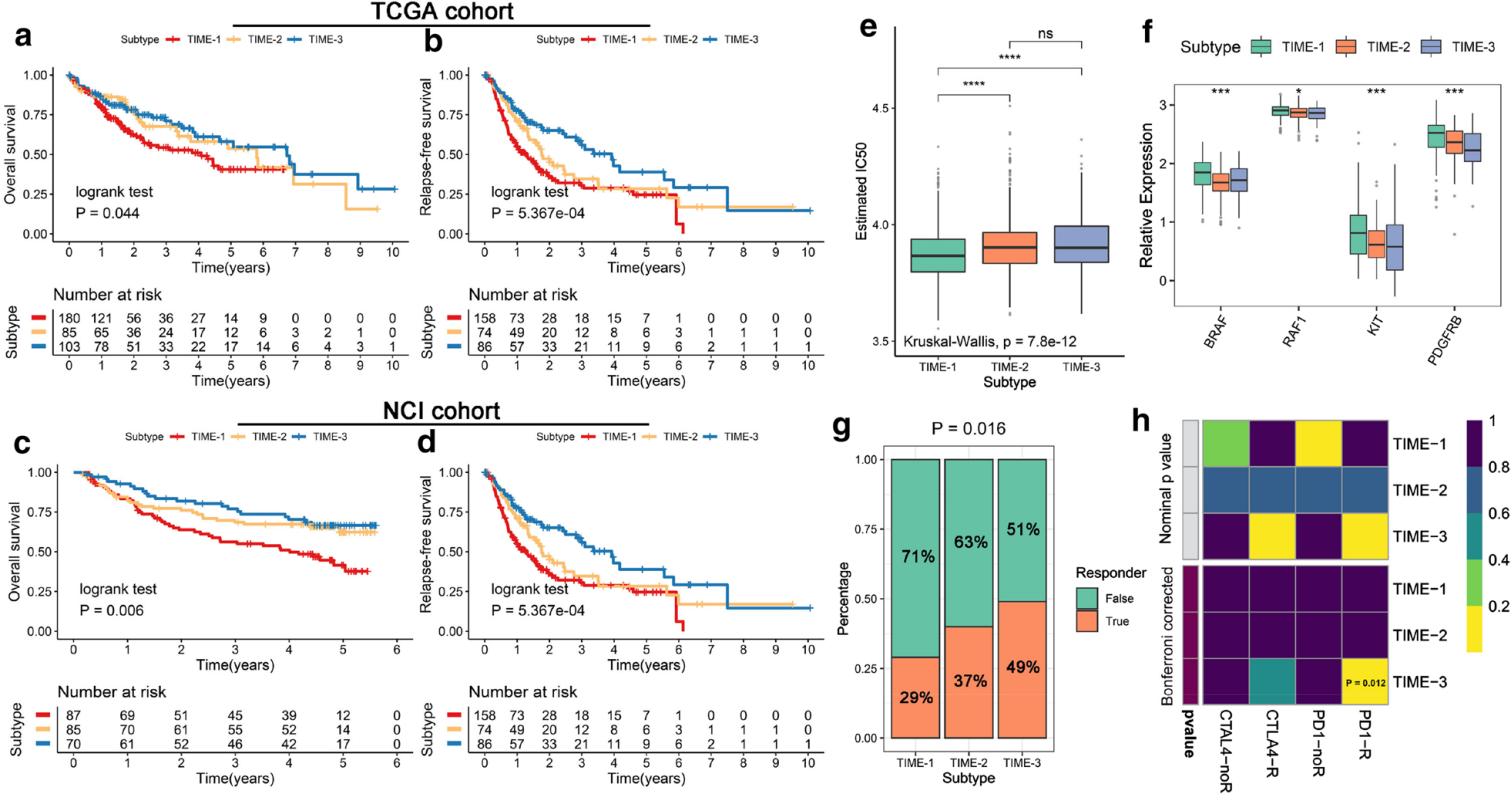
The clinical significance of the TIME phenotypes. **a**, **b** Kaplan–Meier curves for OS (**a**) and RFS (**b**) among three TIME phenotypes in the TCGA cohort. **c**, **d** Kaplan–Meier curves for OS (**c**) and RFS (**d**) among three TIME phenotypes in the NCI cohort. **e** Distribution of the estimated IC50 of sorafenib among three TIME phenotypes in GEO cohort. **f** The expression of sorafenib-related targets in three TIME phenotypes. **g** Distribution of the immunotherapy response results predicted by TIDE algorithm among three TIME phenotypes in the GEO cohort. **h** Submap analysis of the GEO cohort and 47 previous melanoma patients with detailed immunotherapeutic information. For the boxplot, the asterisks represented the statistical p value (*P < 0.05, **P < 0.01, *** P < 0.001, **** P < 0.0001)

We explored the prognostic value in the two independent cohorts (TCGA-LIHC and NCI cohort), which contained the complete overall survival (OS) and relapse free survival (RFS) information. The Kaplan–Meier analysis of both OS and RFS exhibited that HCC patients had an increasingly favorable prognosis from TIME-1 to TIME-3 (Fig. 3a–d). Furthermore, we assessed the sensitivity to sorafenib in TIME phenotypes by the pRRophetic package. TIME-1 was found to be more sensitive to sorafenib than the other phenotypes (Fig. 3e and Additional file 5: Fig. S3A). Notably, TIME-1 exhibited the highest expression in sorafenib related target genes (Fig. 3f). Therefore, this suggested that patients in TIME-1 may benefit from sorafenib the most. As formerly mentioned, TIME-3 had higher levels of immune cell infiltration abundance (e.g. CD8+ T cells). Hence, we speculated that patients in TIME-3 might be more responsive to immunotherapy. The TIDE algorithm was applied to infer the response to immunotherapy. As respected, TIME-3 had a higher response rate than the other phenotypes (Fisher’s exact test: P = 0.009) in the GEO cohort (Fig. 3g), and consistent results was found in the TCGA cohort (Fisher’s exact test: P = 0.048) (Additional file 5: Fig. S3B). We also utilized the submap algorithm to compare the similarity of the expression profiles between the three TIME phenotypes and 47 previous melanoma patients with detailed immunotherapeutic information, and revealed that patients in TIME-3 were more responsive to anti-PD1 treatment (Bonferroni corrected P = 0.008)[30] (Fig. 3h). The submap analysis on the TCGA cohort also achieved similar results (Additional file 5: Fig. S3C).

### Potential extrinsic immune escape mechanism

To further research the regulatory mechanisms of the TIME phenotypes, we focused on the TCGA cohort, which possessed multiple omics data and comprehensive clinical data.

**Fig. 4:**
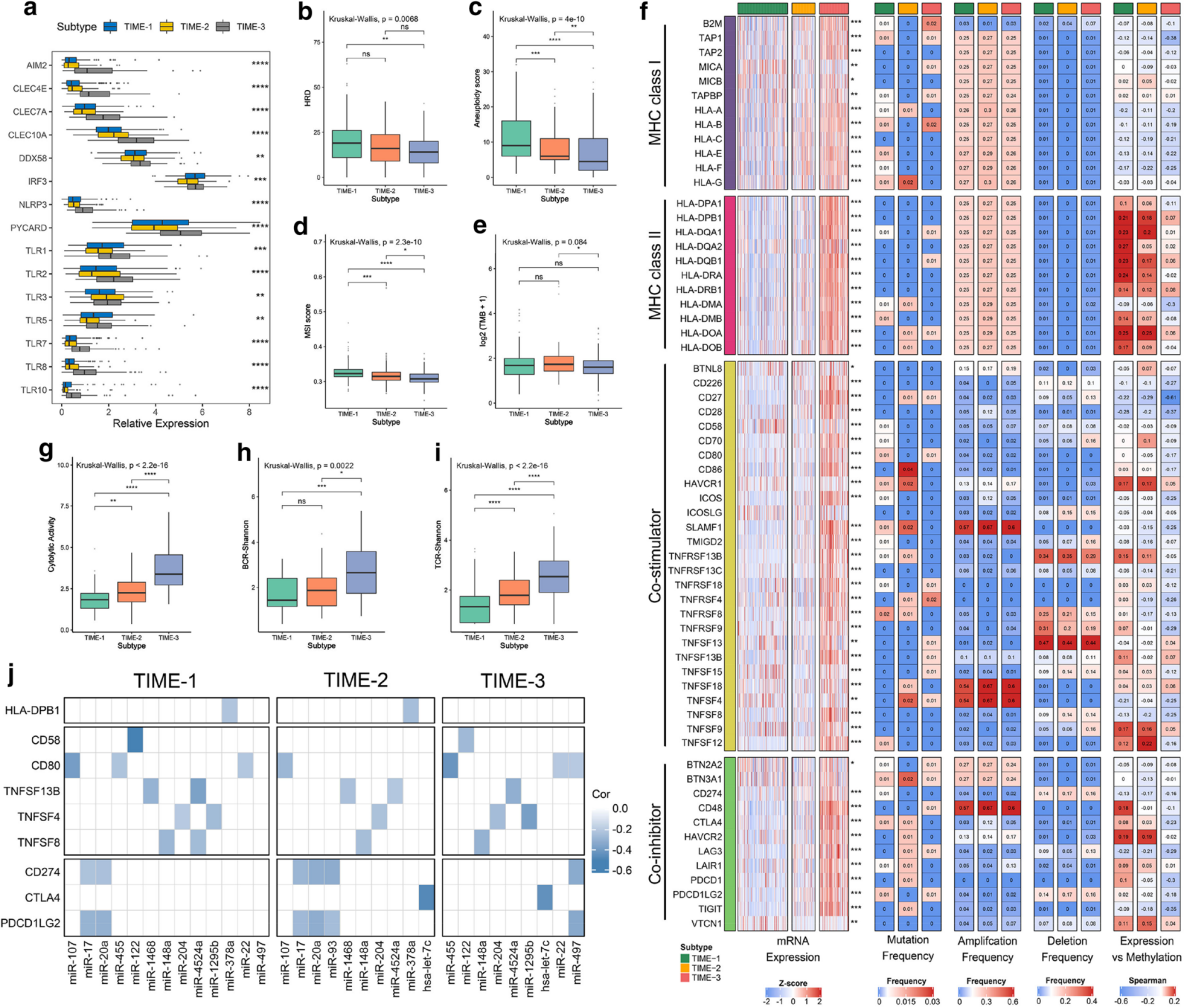
Potential immune escape mechanisms of each phenotype. **a** The mRNA expression of molecules potentially involved in priming of innate immunity. The distribution of HRD (**b**), AS (**c**), MSI score (**d**), and TMB (**e**) in three TIME phenotypes. **f**, From left to right: mRNA expression (z-score), mutation frequency, amplification frequency, deletion frequency, and expression versus methylation (gene expression correlation with DNA-methylation beta-value) for 62 immunomodulators in the TIME phenotypes. The distribution of CYT value (**g**), BCR diversity (**h**), and TCR diversity (**i**) in three TIME phenotypes. **j** Regulation of immunomodulators by miRNA. Associations are shown between commonly implicated miRNAs and immunomodulators for each TIME phenotype. All associations shown represent BH-adjusted p-value < 0.05 and Spearman correlation ≤ -0.2; each miRNA included is negatively correlated with a gene for which it is predicted to bind in miRDB. For the boxplot, the asterisks represented the statistical p value (^ns^P > 0.05, *P < 0.05, **P < 0.01, *** P < 0.001, **** P < 0.0001). For the heatmap, the asterisks represented the statistical p value (*P < 0.05, **P < 0.01, *** P < 0.001)

We firstly investigated the extrinsic immune escape mechanisms. Previous studies indicated that extrinsic immune escape may include three major aspects: absence of leukocytes, presence of immunosuppressive cells, and release of abundant immunosuppressive cytokines [31, 32]. As described above, TIME-1 was characterized by deficient immune cell infiltration and then lack of immune mediated elimination. TIME-2 was characterized by higher levels of immunosuppressive cells (e.g. TH17 cell and Treg; Additional file 6: Fig. S4A, B), which indicated a role of immunosuppressive cells in immune escape. In addition, TIME-2 lacked immune active cells (e.g. CD8+ T cells). Therefore, it was speculated that TIME-1 and TIME-2 probably reflect the absence of recruitment or activation of innate immune cells, inducing failure of adaptive anti-tumor immune responses. The low expression of molecules in TIME-1 and TIME-2, such as AIM2, TLR7 and TLR8, was potentially involved in priming of innate immunity, which further confirmed our speculation (Fig. 4a). TIME-3 was characterized by the presence of abundant innate and adaptive immune cells. In addition, TIME-3 had a higher expression of both immunostimulatory and immunoinhibitory cytokines, while these cytokines were all relatively low in TIME-1 and TIME-2 (Additional file 6: Fig. S4C). These results implied that high concentrations of immunoinhibitory cytokines might contribute to the immune escape in TIME-3. It was noteworthy that the differential expression of cytokines in these three phenotypes could not be explained by the CNV and mutation frequency (all, P > 0.05; Additional file 2: Table S6). Overall, our analysis revealed that the extrinsic immune escape mechanisms of three phenotypes were lack of tumor-infiltrating leukocytes, increased immunosuppressive cells, and rich in immunoinhibitory cytokines, respectively.

### Potential intrinsic immune escape mechanism

We further explored the potential intrinsic immune escape mechanisms in two major facets: tumor immunogenicity and the expression level of immune checkpoint molecules [33]. First, a series of elements associated with tumor immunogenicity were estimated: genomic instability degree, neoantigen burden, genomic viral content, CTA level, and tumor antigen presentation competence [34]. The first four elements were the main source of tumor-specific antigens. It was found that the genomic instability degree presented a decreasing trend from TIME-1 to TIME-3 (HRD, AS and MSI; all, P < 0.05; Fig. 4b–d). Similarly, TIME-3 had a lower TMB, when compared to TIME-2 (P < 0.05, Fig. 4e). In terms of genomic viral content, TIME-3 had more HBV read counts than TIME-1 (mean viral read counts, 44.365 vs. 20.907, P < 0.05; Additional file 6: Fig. S4D), in contrast to the HCV read counts (mean viral read counts, 12.931 vs. 16.111, P < 0.1; Additional file 6: Fig. S4E). Of note, the neoantigen burden was relatively lower in TIME-3, although the statistical difference among the three phenotypes in SNV or indel neoantigens was not reached (Additional file 6: Fig. S4F, G). There was also not distinct variation among the CTAs overall expression of the three phenotypes (Additional file 6: Fig. S4H). Overall, these above indicators had little difference in the TIME phenotypes. We further investigated the tumor antigen presentation capacity of the three phenotypes, and observed that TIME-3 had the highest APS and MHC-related molecules expression level, as opposed to TIME-1 (all, P < 0.01; Additional file 6: Fig. S4I and Fig. 4f), which was consistent with the CYT value and BCR/TCR diversity (all, P < 0.05; Fig. 4g–i and Additional file 6: Fig. S4J, K). This indicated that defective tumor antigen presentation capacity may be an intrinsic immune escape mechanism for TIME-1.

Subsequently, the genomic alterations of 62 immunomodulators were further summarized within the three TIME phenotypes (Fig. 4f). It was found that TIME-3 had higher costimulatory and coinhibitory molecules than the other phenotypes. This suggested that TIME-3 may overexpress the immune checkpoint molecules (such as CTLA4, CD274 and PDCD1; all BH-adjusted P < 0.001) to evade the immune elimination after immune activation. All somatic mutations and CNVs did not significantly differ among the three phenotypes, and most of immunomodulators exhibited rare somatic mutations and CNVs, which indicated that the mutations and CNVs in the immunomodulators had little effect on TIME. Of note, DNA methylation negatively regulated many immunomodulators, such as CD27, CD226 and TNFSF8, implying epigenetic silencing. The associations were shown between miRNAs and immunomodulators for each TIME phenotype (BH corrected P-value < 0.05; Fig. 4j), such as miR-17 negative correlated with CD274 and PDCD1LG2. It was also observed that the three phenotypes shared a common TNFSF4 negative regulator: miR-204. Compared with the mutation and CNV, methylation modification and miRNA sponges played leading roles in regulating the immunomodulators, indicating a new perspective for the development of immune checkpoint inhibitors.

### Genomic alterations of the three TIME phenotypes

**Fig. 5:**
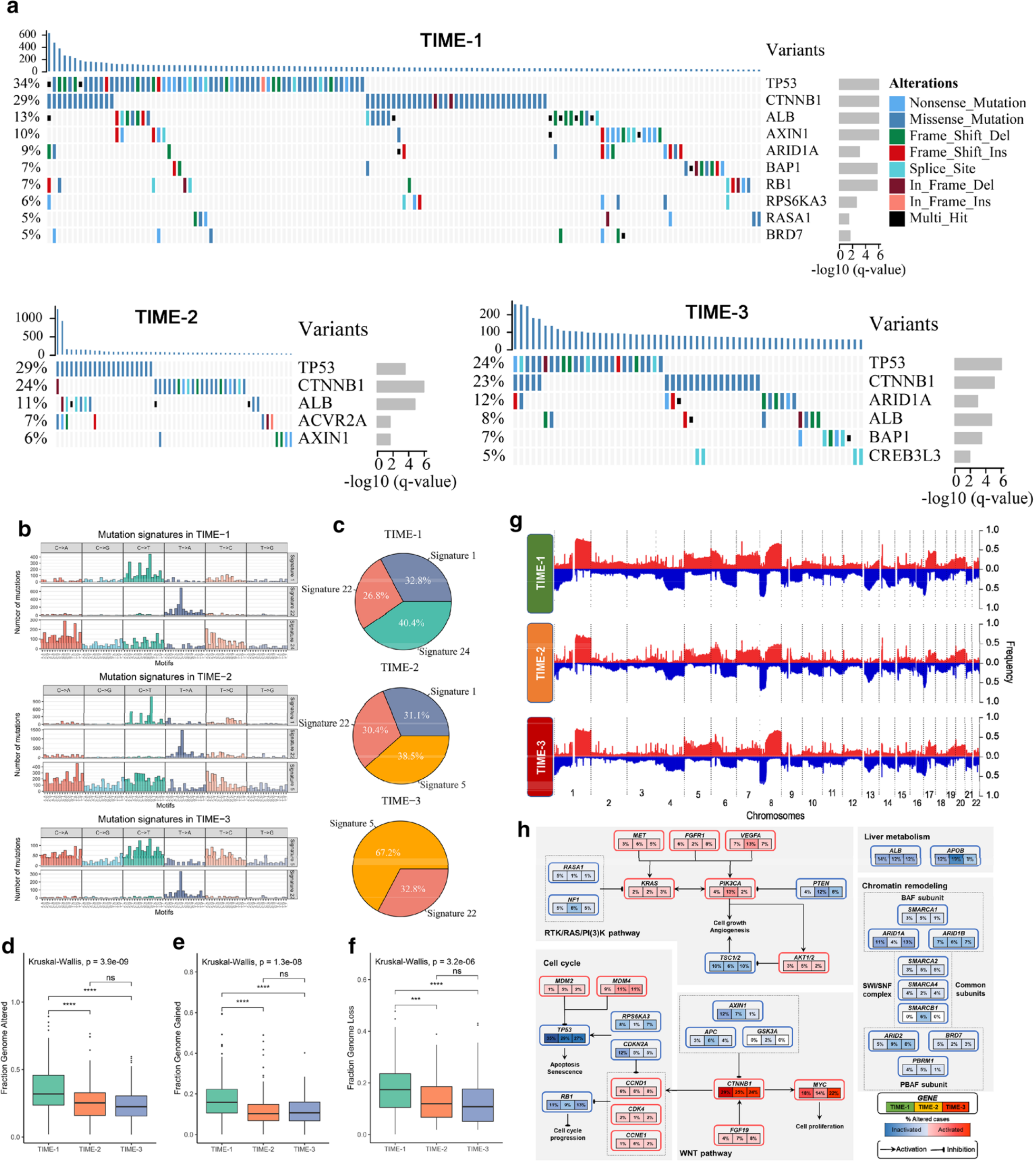
Genomic alterations of the TIME phenotypes. **a** Significantly mutated genes (SMGs) in three TIME phenotypes. **b** Mutation signatures extracted from three TIME phenotypes. **c** The pie chart shows the proportion of mutation signatures in three TIME phenotypes. The distribution of FGA (**d**), FGG (**e**) and FGL (**f**) in three TIME phenotypes. **g** Gain (red) or loss (blue) frequencies of copy number variations (CNVs) in the autosomes of HCC patients. **h** Integrated molecular comparison of genomic alterations in signaling pathways across the TIME phenotypes. Each gene box includes three percentages representing the frequency of activation or inactivation in TIME-1, 2, and 3. All changes are tallied together in calculating the percentages of altered cases within each TIME phenotype. Genomic alterations include mutations and copy-number changes. Missense mutations are only counted if they have known oncogenic function, have been reported in COSMIC, or occur at known mutational hotspots. Genes are grouped by signaling pathways, with edges showing pairwise molecular interactions. For the boxplot, the asterisks represented the statistical p value (^ns^P > 0.05, *P < 0.05, **P < 0.01, *** P < 0.001, **** P < 0.0001)

We separately determined the SMGs among the three phenotypes using MutSigCV (Fig. 5a). All SMGs had mutation rates greater than 5% in three phenotypes. Among these three TIME phenotypes, the common SMGs (including TP53, CTNNB1 and ALB) had the top three significant MutSigCV q-value and frequent mutation rates, indicating that the mutation of TP53, CTNNB1 and ALB was broad in HCC. Additionally, the three phenotypes also displayed distinct SMGs, such as RB1, ACVR2A and CREB3L3 were SMGs of the three phenotypes, respectively. Besides, two newly identified SMGs, namely, BRD7 and RASA1, were classified as tumor suppressor genes, and these were associated with chromosome remodeling and cell proliferation [35, 36]. Based on the NMF, we isolated the mutation signatures of each phenotype. The age-related mutational processes (spontaneous deamination of 5-methylcytosine for signature 1 and unknow aetiology for signature 5) were prevalent in three phenotypes (Fig. 5b). TIME-1 had the least proportion than the other phenotypes (Fig. 5c). In addition, the three phenotypes also shared a common mutation profile (signature 22) associated with exposures to aristolochic acid. This may be associated with high-proportioned Asian patients in three phenotypes (Additional file 7: Fig. S5A), and the aristolochic acid was mainly derived from herbal drugs of traditional Asian medicine [37]. Notably, signature 24, which represented the mutational pattern related with aflatoxin, was identified only in TIME-1, and possessed the maximum proportion (40.4%) (Fig. 5c). This implied that HCC patients in TIME-1 were more likely to be exposed to aflatoxin.

Tumor ploidy was estimated by ABSOLUTE, suggesting that a larger scale of HCC presented genome doubling, and that the doubling pattern was more frequent in TIME-1 compared with the other phenotypes (P = 0.018, Additional file 7: Fig. S5B). As shown in Fig. 4d–f, the FGA, FGG and FGL in TIME-1 were significantly higher than the other phenotypes, which might promote the cell proliferation and immune escape [38]. We further applied GISTIC 2.0 to delineate the significant focal copy number alterations of each phenotype (Fig. 5g and Additional file 2: Table S7). CNVs that were recurrent in TIME-1 contained the focal amplification of 8q24.21 (MYC, ANXA13) and 13q34 (CDC16, TFDP1), and the focal loss of 14q22.1 (SAV1). Recurring focal arms CNVs in TIME-2 included the only amplification of 6p21.1 (VEGFA), and the loss of 13q13.3 (CCNV1). The genes on the focal loss arms of TIME-1 and TIME-2 were mainly associated with chemokines and cytokine through the GO annotation. Hence, the loss of these genes may contribute to the low immune infiltration of TIME-1 and TIME-2. TIME-3 exhibited the focal amplification that involved 8q24.12 (MTBP) and the focal deletion that involved 5q13.2 (TERT) and 10q23.31 (PTEN). These phenotype-special CNVs may play a crucial role in the biological features of the three phenotypes.

Furthermore, the combination of mutation and CNV data revealed the frequent alterations in different pathways (Fig. 5h). It was found that some genome alterations distributed evenly in these three phenotypes, such as TP53, CTNNB1 and ALB. In addition to these common alterations, we also observed the diverse alteration patterns in the pathways among three phenotypes. In TIME-1, cell cycle regulatory factor CDKN2A mutated in 12% of the cases. In TIME-2, VEGFA and its downstream genes PIC3KA and PTEN were frequently altered, and all of which were known to activate the PI3K pathway. APOB consumes an abundant cellular energy to facilitate very low-density lipoprotein (VLDL) secretion, and we found that APOB was frequently altered in 19% of the cases for TIME-2. Overall, the diverse genomic alteration preferences in three phenotypes might contribute to shape the TIME, and lead to differences in immune cell infiltration.

### Methylation modification and regulation of the TIME phenotypes

**Fig. 6:**
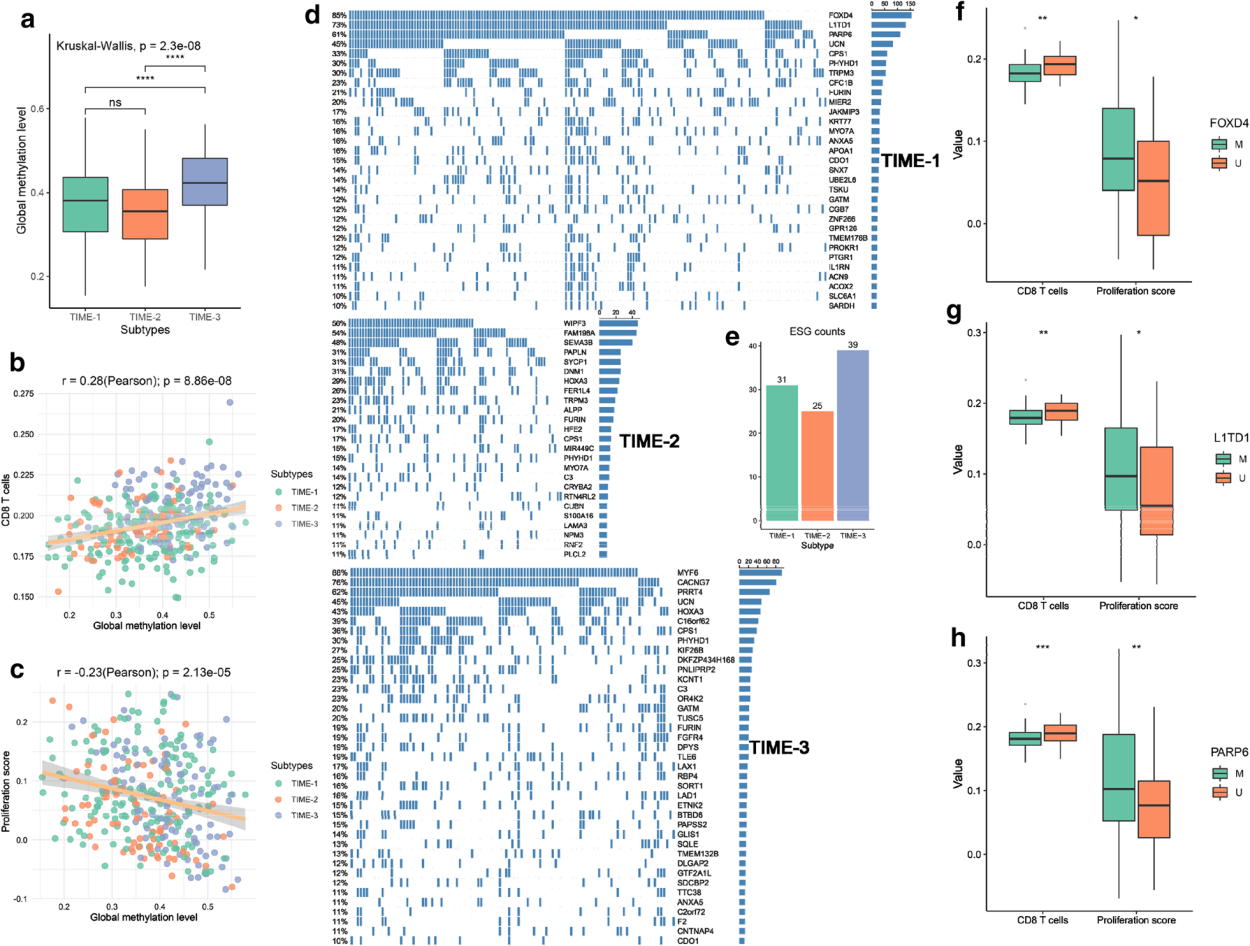
Methylation modification and regulation of the TIME phenotypes. **a** The distribution of global methylation level in three TIME phenotypes. Correlation of GML with CD8 T cells (**b**) and proliferation score (**c**). **d** The epigenetically silenced genes (ESGs) in three TIME phenotypes. Correlation of global methylation level (GML) with CD8 T cells and proliferation score. **e** The number of ESGs in three TIME phenotypes. **f** Differences in CD8 T cells and proliferation scores between FOXD4 methylated and FOXD4 unmethylated groups. **g** Differences in CD8 T cells and proliferation scores between L1TD1 methylated and L1TD1 unmethylated groups. **h** Differences in CD8 T cells and proliferation scores between PARP6 methylated and PARP6 unmethylated groups. For the boxplot, the asterisks represented the statistical p value (^ns^P > 0.05, *P < 0.05, **P < 0.01, *** P < 0.001, **** P < 0.0001)

As tumor cells divide, the loss of global methylation levels (GMLs) can result in chromosomal instability, and affect immune cell infiltration [39–41]. The GMLs in TIME-3 was significantly higher than the other phenotypes (P < 0.001, Fig. 6a). Furthermore, there was a positive correlation between tumor-infiltrating CD8+ T cells and GMLs (Fig. 6b). In contrast, the decrease in GML might promote tumor cell proliferation (Fig. 6c). Subsequently, we identified 31, 25 and 39 ESGs in TIME-1, TIME-2 and TIME-3, respectively (Additional file 2: Table S8 and Fig. 6d, e). It was noted that CPS1, FURIN and PHYHD1 were shared by the three phenotypes. As a liver-specific enzyme, CPS1 can facilitate cell division by instituting the pyrimidine synthesis pathway [42]. In TIME-1, FOXD4 was marked as epigenetically silenced in 85% of the cases, and its methylation silencing may lead to immune system dysfunction and tumor proliferation [43] (Fig. 6f). Tumor suppressors L1TD1 and PARP6, also the specific ESGs of TIME-1, and their methylation may promote tumor proliferation and low immune infiltration [44, 45] (Fig. 6g, h). SEMA3B belonged to the ESG of TIME-2, and its methylation can accelerate the progression of HCC [46]. FER1L is an another ESG of TIME-2, which can activate the PI3K/AKT pathway, further leading to the formation of the immunosuppressive microenvironment [47]. These results suggested that ESGs exhibit diversity among the three phenotypes, which may be involved in shaping the TIME, and these specific ESGs may also be potential therapeutic targets.

### A robust prognostic and immunotherapy signature: TIME index

**Fig. 7:**
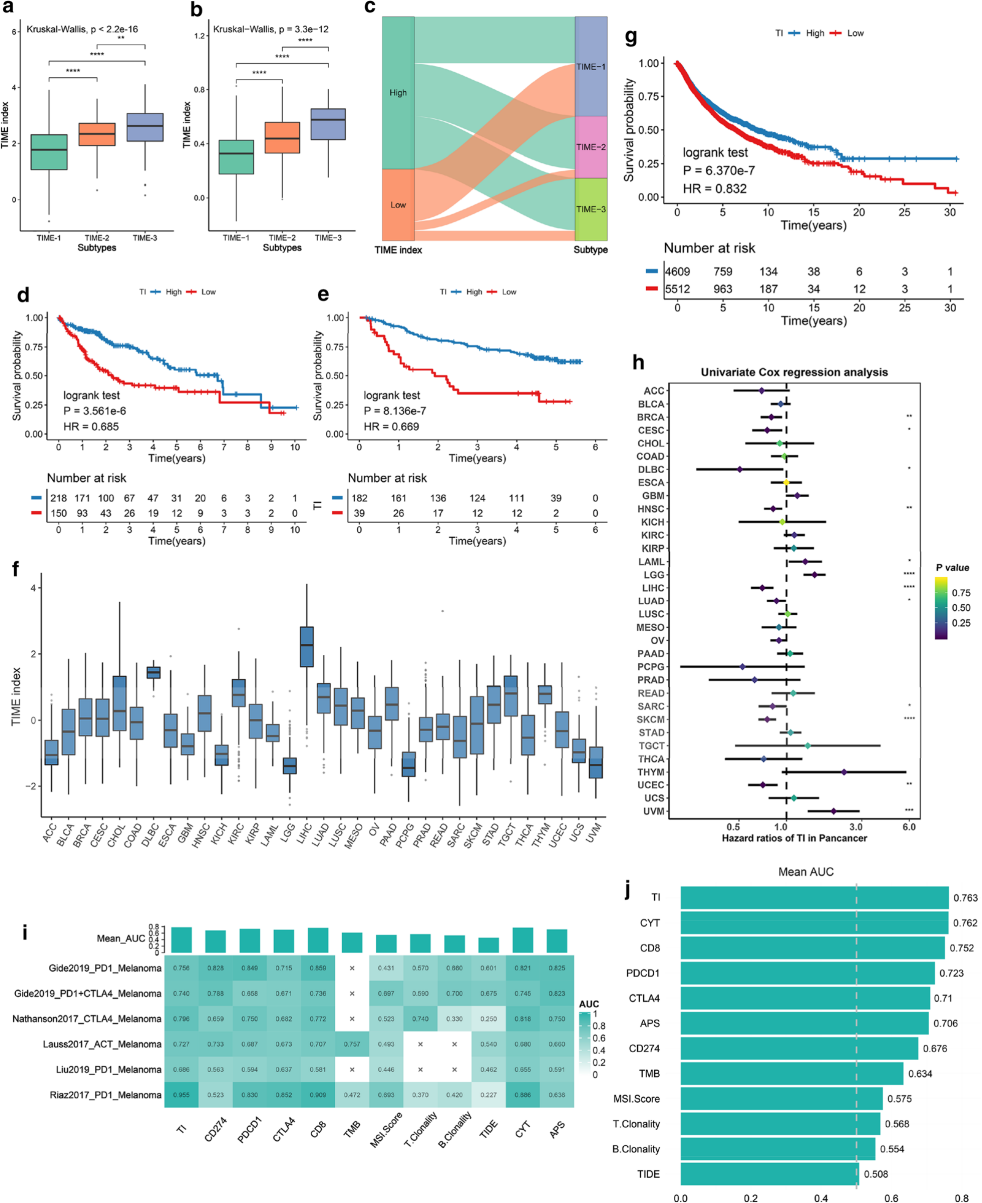
The distribution and clinical significance of TIME index (TI). The TI distribution of three TIME phenotypes in the TCGA cohort (**a**) and NCI cohort (**b**). **c** Alluvial diagram showing the changes of TI and the three TIME phenotypes. Kaplan–Meier curves for OS of HCC patients in the TCGA cohort (**d**) and NCI cohort (**e**). **f** The TI distribution in 33 cancer types. **G** Kaplan–Meier curves for OS of pancancer patients. **h** The univariate Cox’s regression result of TI in 33 cancer types. **i** The accuracy of TI and 11 other biomarkers in predicting immunotherapy, each cell represents the corresponding AUC value of one biomarker in one cohort. **j** The mean AUC of TI and 11 other biomarkers. For the boxplot, the asterisks represented the statistical p value (^ns^P > 0.05, *P < 0.05, **P < 0.01, *** P < 0.001, **** P < 0.0001)

We identified 98 phenotype-related DEGs (Additional file 2: Table S9). These genes, which significantly varied within the three phenotypes, possibly contributed to form the heterogenous TIME of HCC. In addition, many genes have been reported to be critical in immune response, such as CD27, CD8A, GZMA and IL7R [48–51]. The GO and KEGG annotation also displayed intensive immune phenotypes (Additional file 2: Table S10, S11, Additional file 8: Fig. S6A, B). Based on these DEGs, the ssGSEA was performed to obtain the TI of each patient. The TI presented a gradual increase from TIME1 to TIME3 (Fig. 7a, b). HCC patients with low TI were mainly distributed in TIME-1 (Fig. 7C). According to the optimal cut-off determined by the survminer package, we classified HCC patients into high and low TI groups. As expected, patients in the high TI group exhibited a tendency to better outcomes in the two independent cohort (Log-rank P < 0.001, Fig. 7d, e). Besides, the multivariable Cox regression revealed that the TI was an independently prognostic factor in HCC (P = 0.018, Additional file 2: Table S12). We further assessed the TI of 10,121 patients that involved 33 differing types of cancers. Obvious TI diversity was observed in different cancers (Fig. 7f), which indicated that heterogeneous immune infiltration existed not only within the tumor, but also between tumors. The survival analysis for pancancer indicated that patients with a higher TI had a better prognosis (Log-rank P < 0.001, Fig. 7g) and that the TI could independently affect the prognosis in the multivariable Cox regression (P = 0.006, Additional file 2: Table S12). Furthermore, it was found that 24 of 33 cancers presented a statistical significance in the Kaplan–Meier analysis (Additional file 9: Fig. S7), and univariate Cox’s regression indicated that the TI was a protective factor in many different tumors (Fig. 7h).

We further explored the predictive ability of the TI for immunotherapy. In order to determine the predictive power, we also computed the response prediction of 11 other known biomarkers. The area under the ROC curve (AUC) was used as the quality metric of prediction. We found that the TI exhibited robust predictions in the six cohorts (Fig. 7i). Especially in the Riaz et al. cohort, the TI reached the highest prediction accuracy with AUC = 0.955. It was also observed that although the AUC value of TI in the Liu et al. cohort was less than 0.7, this was still the highest, when compared to the other markers. In addition, the TI displayed the highest mean AUC value compared with the other biomarkers (Fig. 7j). Hence, the present work strongly suggests that the TI was a potential and robust biomarker for the prognosis and clinical response assessment of immunotherapy.

In addition, to advance clinical application, we developed a R package termed “TIME” in the GitHub website (https://github.com/Zaoqu-Liu/TIME). The pipeline could classify single patient into one of these three subtypes and calculated the TI.

## Discussion

The hepatocellular carcinoma (HCC) ecosystem is diverse, complex and dynamic in nature, and is mainly composed of tumor cells and immune cells [52]. Immune cells are the main components of TIME, and their number and status play a crucial role in the progression of tumor development, invasion and metastasis. To the best of our knowledge, the present study is the first to systematically investigate the heterogeneity of TIME from the dimension of broad-spectrum immune cells, and comprehensively explore the potential immune escape mechanisms and specific genomic alterations of different TIME phenotypes. In addition, the TI was proposed to quantify TIME infiltration pattern, and it was also a superior prognostic and immunotherapy predictor. These results can enhance the understanding of TIME, and guide more effective personalized immunotherapies.

As described, TIME-1 was characterized by immune cell depletion and proliferation, corresponding to the immune-deficiency phenotype, TIME-2 was characterized by enrichment of immunosuppressive cells, corresponding to the immune-suppressed phenotype, and TIME-3 was characterized by abundant immune cell infiltration and immune activation, corresponding to immune-activated phenotype. We underlined the potential immune escape mechanism of each phenotype: lack of leukocytes and defective tumor antigen presentation capacity in TIME-1, increased immunosuppressive cells in TIME-2, and rich in immunoinhibitory molecules in TIME-3. The distinct immune escape mechanism among TIME phenotypes could provide strategies for improving the efficacy of HCC immunotherapy. In addition, it was also found that methylation modification and miRNA sponges may play leading roles in regulating the immunomodulator expression, when compared to mutations and CNVs, suggesting that the development of ICIs should consider the methylation modification and miRNA regulation of immunomodulators.

The TIME phenotypes also exhibited a significant heterogeneity at the genomic level, which may drive the formation of different phenotypes. TIME-1 was dominated by the alterations of TP53, CDKN2A, CTNNB1 and AXIN1. CDKN2A could inhibit the tumor-promoting behavior of CDK4/6, and given that the CDKN2A alteration is frequent in HCC, CDK4/6 inhibitors are presently being tested in advanced HCC [53]. Thus, it was suspected that TIME-1 may be more sensitive to CDK4/6 inhibitors. Previous studies showed that the mutations of CTNNB1 and AXIN1 might be characteristic of immune exclusion, and represent the biomarkers of innate resistance to immunotherapy [54, 55]. Besides, the FOXD4 methylation was also associated with the immune dysfunction and cell proliferation. These genomic events might contribute to the immunodeficiency and proliferation of TIME-1. TIME-2 was characterized by significant alteration patterns in the PI3K pathway, such as PIK3CA, VEGFA and PTEN. The mutation of PIK3CA may serve as a reliable biomarker for relatively poor response to immunotherapies, such as PD-L1 antibodies [56]. VEGFA, as a tumor angiogenic factor, also plays a pivotal role in the formation of the tumor immunosuppressive microenvironment. Its alterations induce the proliferation of TH17 and Treg, and inhibit CD8 + T cell function, resulting in immune escape [57–59], which in line with the immune cell infiltration characteristic of TIME-2. Of note, TIME-2 might be more sensitive to the VEGFA-targeting monoclonal antibody Bevacizumab due to its significant VEGFA amplification [60]. ARID1A was the SMG of TIME-3, and its inactivating mutations can lead to remarkable increases in CD8 and PD-L1, and tumors with ARID1A deficiency were also more sensitive to PD-L1 antibodies [61]. Overall, these results suggested that distinct genomic alterations might not only lead to different immune cell infiltration and functional status, but also explain the potential reasons for the sensitivity or resistance of different phenotypes to immunotherapy, which provide references for the precise treatment of HCC.

The TIME phenotypes have significant clinical value. Consistent with the immune infiltration of three phenotypes, TIME-1 indicated the worst prognosis, while TIME-3 had the most favorable prognosis. TIME-1 was predicted to be most sensitive to sorafenib, which was consistent with higher expression of drug targets. Unsurprisingly, in line with the higher level of immune cells infiltration and immune checkpoint molecule expression, TIME-3 exhibited a superior response to immunotherapy. In addition, we proposed a scoring scheme to quantify the TIME infiltration pattern termed TIME index (TI). The TI was not only an independent prognostic biomarker for both HCC and pancancer, but also performed well in predicting the response to immunotherapy. Hence, the TI could guide clinical management and personalized immunotherapy of HCC. In addition, for the purpose of facilitating clinical application, we developed a pipeline to classify single patient into one of these three subtypes and calculated the TI.

The present study also had some limitations. First, we only considered the inter-individual heterogeneity due to the lack of data, but did not consider the intra-tumor heterogeneity, which is common in multifocal HCC [62]. Second, although machine learning algorithms were applied to predict the sensitivity of the TIME phenotypes to sorafenib and immunotherapy, further clinical validation is need. Finally, potential genomic drivers require further functional verification. Clinical studies and related experiments are ongoing in our hospitals and laboratories.

## Conclusions

In summary, our research revealed the three heterogeneous TIME phenotypes with different clinical outcomes, immune escape mechanisms, and genomic alterations in HCC, which could present strategies for improving the efficacy of immunotherapy. The TI as a novel prognostic and immunotherapeutic signature that could guide clinical management and personalized immunotherapy.

## Supplementary Information

**Additional file 1: Fig. S1.** The immune infiltration pattern of the TIME phenotypes. **A**, Association between 24 immune cell subsets. **B**, Recommended number of clusters using 26 criteria of Nbclust package in the GEO cohort. **C**, The immune cells infiltration pattern assessed by CIBERSORT algorithm. **D**, The immune cells infiltration pattern assessed by MCP-counter algorithm. **E**, The infiltration abundance of 24 immune cell subsets evaluated by ssGSEA algorithm for three TIME phenotypes in TCGA cohort. Survival status, age, gender, vascular invasion, histology grade, AJCC stage, the TIME phenotypes are shown as patient annotations. **F**, The differences of 24 immune cell subsets infiltration among the three TIME phenotypes in the TCGA cohort. G, Recommended number of clusters using 26 criteria of Nbclust package in the TCGA cohort. For the boxplot, the asterisks represented the statistical p value (*P < 0.05, **P < 0.01, *** P < 0.001, **** P < 0.0001).

**Additional file 2: Table S1.** Basic information of datasets included in this study for identifying distinct TIME phenotypes. **Table S2.** The specific Hallmark pathways of each TIME phenotype in the GEO cohort. **Table S3.** The specific KEGG pathways of each TIME phenotype in the GEO cohort. **Table S4.** The specific Hallmark pathways of each TIME phenotype in the TCGA cohort. **Table S5.** The specific KEGG pathways of each TIME phenotype in the TCGA cohort. **Table S6.** The difference of chemokines, ILs, IFNs, and other important cytokines and their receptors among three TIME phenotypes in the expression, CNV and mutation level. **Table S7.** The significant focal copy number alterations (including amplification and deletion) of each TIME phenotype. **Table S8.** The epigenetically silenced genes (ESGs) in three TIME phenotypes. **Table S9.** Ninety-eight phenotype-related differentially expressed genes (DEGs). **Table S10.** GO enrichment analysis of 98 phenotype-related DEGs. **Table S11.** KEGG enrichment analysis of 98 phenotype-related DEGs. **Table S12.** Multivariate COX regression analysis of TI in HCC and pancancer.

**Additional file 3.** Materials and methods.

**Additional file 4: Fig. S2.** The specific functional status and biological characteristics of each TIME phenotype in the TCGA cohort. **A**, The activation states of Hallmark pathways of distinct TIME phenotypes in the TCGA cohort. **B**, The activation states of KEGG pathways of distinct TIME phenotypes in the TCGA cohort.

**Additional file 5: Fig. S3.** Assessment of chemotherapy and immunotherapy in the TCGA Cohort. **A**, Distribution of the estimated IC50 of sorafenib among three TIME phenotypes in the TCGA cohort. **B**, The distribution of the immunotherapy response results predicted by TIDE algorithm among three TIME phenotypes in the TCGA cohort. **C**, Submap analysis of the TCGA cohort and 47 previous melanoma patients with detailed immunotherapeutic information. For the boxplot, the asterisks represented the statistical p value (*P < 0.05, **P < 0.01, *** P < 0.001, **** P < 0.0001).

**Additional file 6: Fig. S4.** Potential immune escape mechanisms of each phenotype. **A**, **B**, The difference of TH17 (**A**) and Treg (**B**) among the three TIME phenotypes. **C**, The mRNA expression of chemokines, interleukins, interferons, and other important cytokines and their receptors for each TIME phenotype. The distribution of HBV read counts (**D**), HCV read counts (**E**), SNV neoantigens (**F**), indel neoantigens (**G**), CTA score (**H**), APS value (**I**), BCR-Richness diversity (**J**) and TCR-Richness diversity (**K**) in three TIME phenotypes. For the boxplot, the asterisks represented the statistical p value (nsP > 0.05, *P < 0.05, **P < 0.01, *** P < 0.001, **** P < 0.0001). For the heatmap, the asterisks represented the statistical p value (*P < 0.05, **P < 0.01, *** P < 0.001, **** P < 0.0001).

**Additional file 7: Fig. S5.** The distribution of race and ploidy. A, The distribution of race in the TCGA cohort. B, The distribution of tumor ploidy in three TIME phenotypes.

**Additional file 8: Fig. S6.** GO and KEGG enrichment analysis of differentially expressed genes (DEGs).

**Additional file 9: Fig. S7.** Kaplan–Meier curves of OS for the TIME index (TI) in the 33 cancer types.

## Data Availability

All data used in this work can be acquired from the Gene-Expression Omnibus (GEO; https://www.ncbi.nlm.nih.gov/geo/) and the GDC portal (https://portal.gdc.cancer.gov/).

## Abbreviations

TIME: Tumor immunological microenvironment
HCC: Hepatocellular carcinoma
TI: TIME index
GEO: Gene Expression Ominibus
ssGSEA: Single sample gene set enrichment analysis
CDF: Cumulative distribution function
PAC: Proportion of ambiguous clustering
IGP: In-group proportion
GSVA: Gene set variation analysis
TMB: Tumor mutation burden
AS: Aneuploidy scores
HRD: Homologous recombination defects
MSI: Microsatellite instability
CTAs: Cancer/testis-antigens
APS: Antigen processing and presenting machinery scores
CNV: Copy number variation
SMGs: Significantly mutated genes
NMF: Non-negative matrix factorization
FGA: Fraction of genome alteration
FGG: Fraction of genome gained
FGL: Fraction of genome lost
GML: Global methylation level
ESGs: Epigenetically silenced genes
DEGs: Differentially expressed genes
OS: Overall survival
RFS: Relapse free survival
CYT: Cytolytic activity
VLDL: Very low-density lipoprotein
AUC: Area under the ROC curve
