# Supplementary figures and images for: A novel immune classification reveals distinct immune escape mechanism and genomic alterations: implications for immunotherapy in hepatocellular carcinoma
Source: J Transl Med. 2021 Jan 6;19:5. doi: 10.1186/s12967-020-02697-y (PMC7789239; doi:10.1186/s12967-020-02697-y)

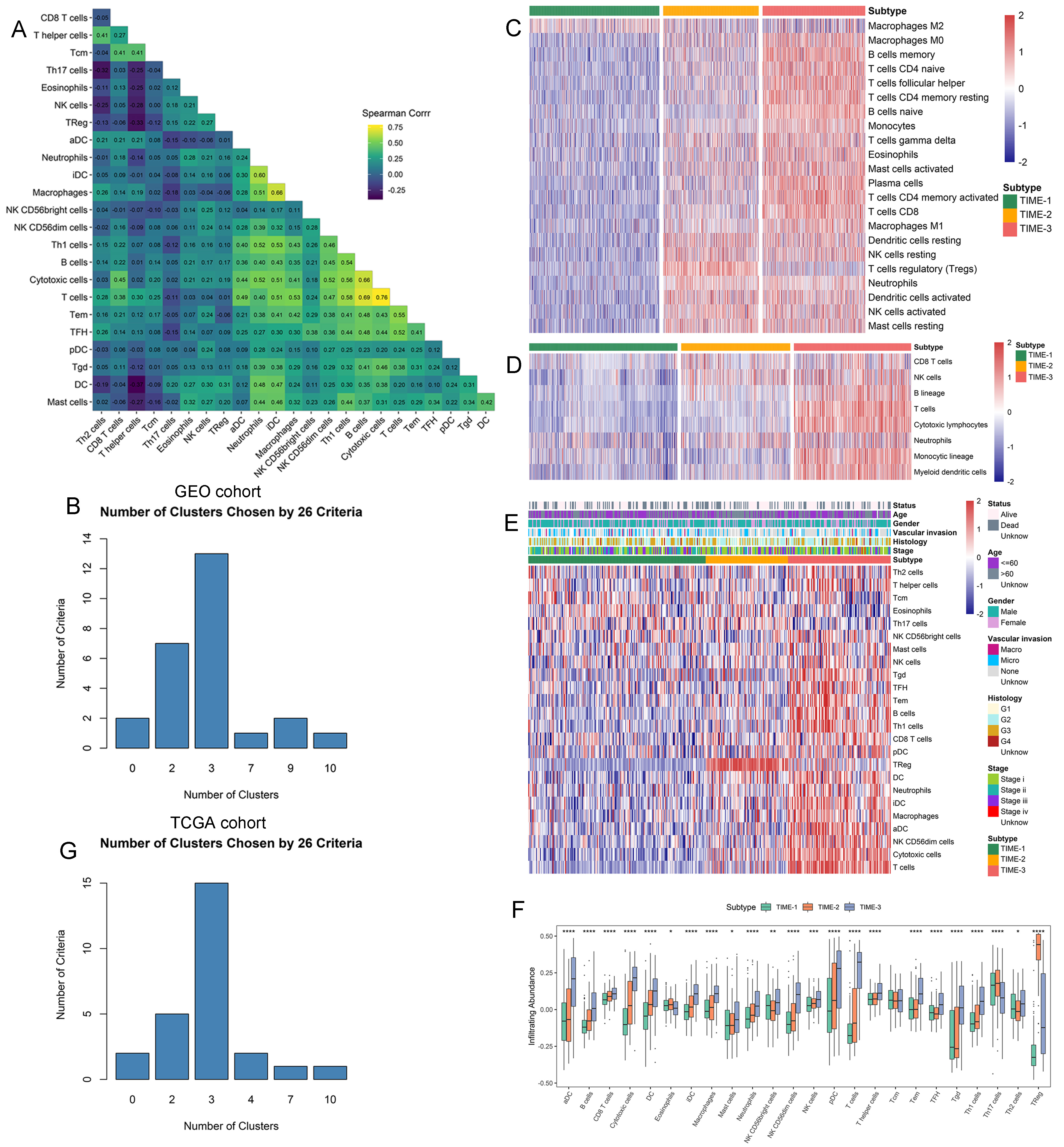

Supplement: Supplementary file 1 — Additional file 1: Fig. S1. The immune infiltration pattern of the TIME phenotypes. A, Association between 24 immune cell subsets. B, Recommended number of clusters using 26 criteria of Nbclust package in the GEO cohort. C, The immune cells infiltration pattern assessed by CIBERSORT algorithm. D, The immune cells infiltration pattern assessed by MCP-counter algorithm. E, The infiltration abundance of 24 immune cell subsets evaluated by ssGSEA algorithm for three TIME phenotypes in TCGA cohort. Survival status, age, gender, vascular invasion, histology grade, AJCC stage, the TIME phenotypes are shown as patient annotations. F, The differences of 24 immune cell subsets infiltration among the three TIME phenotypes in the TCGA cohort. G, Recommended number of clusters using 26 criteria of Nbclust package in the TCGA cohort. For the boxplot, the asterisks represented the statistical p value (*P < 0.05, **P < 0.01, *** P < 0.001, **** P < 0.0001). [file 12967_2020_2697_MOESM1_ESM.jpg]

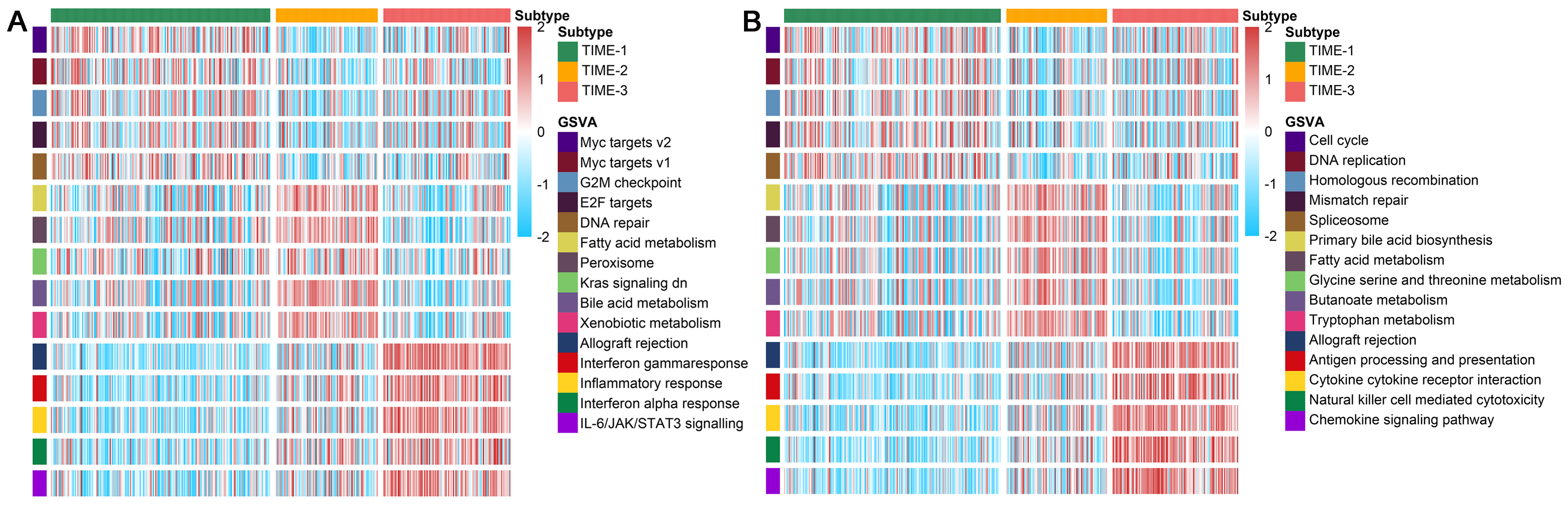

Supplement: Supplementary file 4 — Additional file 4: Fig. S2. The specific functional status and biological characteristics of each TIME phenotype in the TCGA cohort. A, The activation states of Hallmark pathways of distinct TIME phenotypes in the TCGA cohort. B, The activation states of KEGG pathways of distinct TIME phenotypes in the TCGA cohort. [file 12967_2020_2697_MOESM4_ESM.jpg]

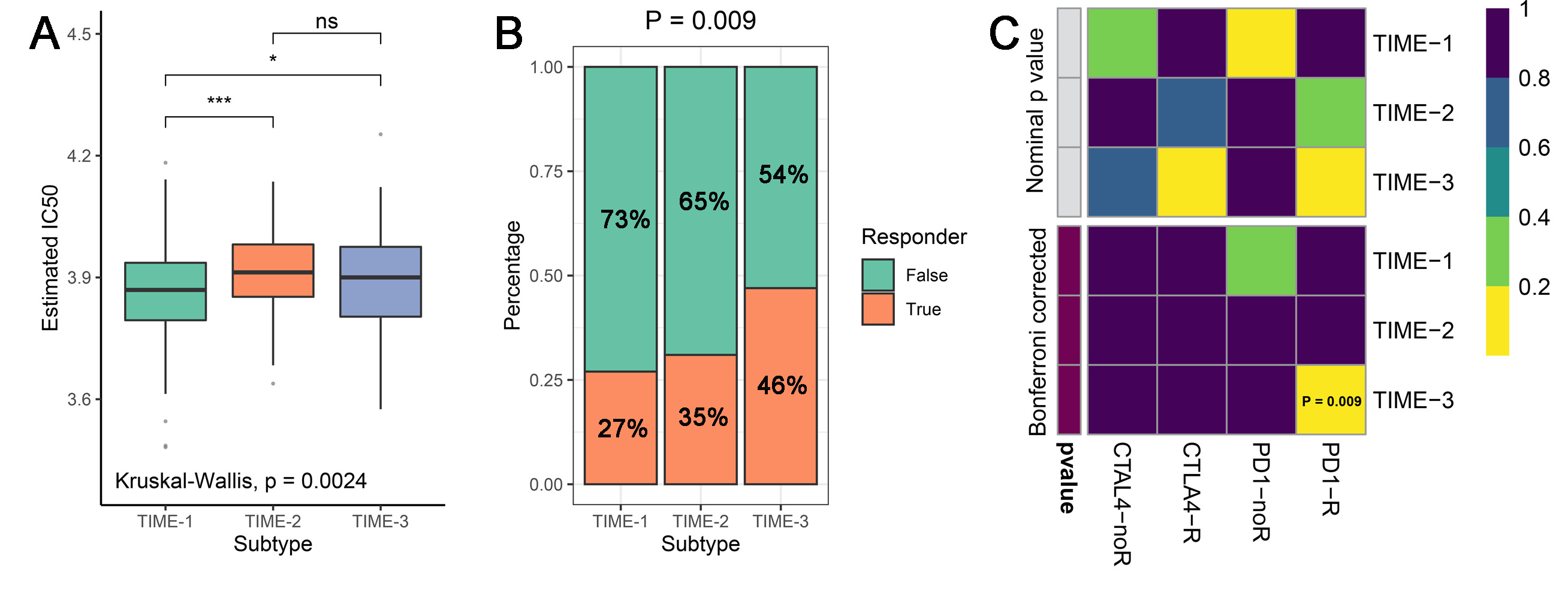

Supplement: Supplementary file 5 — Additional file 5: Fig. S3. Assessment of chemotherapy and immunotherapy in the TCGA Cohort. A, Distribution of the estimated IC50 of sorafenib among three TIME phenotypes in the TCGA cohort. B, The distribution of the immunotherapy response results predicted by TIDE algorithm among three TIME phenotypes in the TCGA cohort. C, Submap analysis of the TCGA cohort and 47 previous melanoma patients with detailed immunotherapeutic information. For the boxplot, the asterisks represented the statistical p value (*P < 0.05, **P < 0.01, *** P < 0.001, **** P < 0.0001). [file 12967_2020_2697_MOESM5_ESM.jpg]

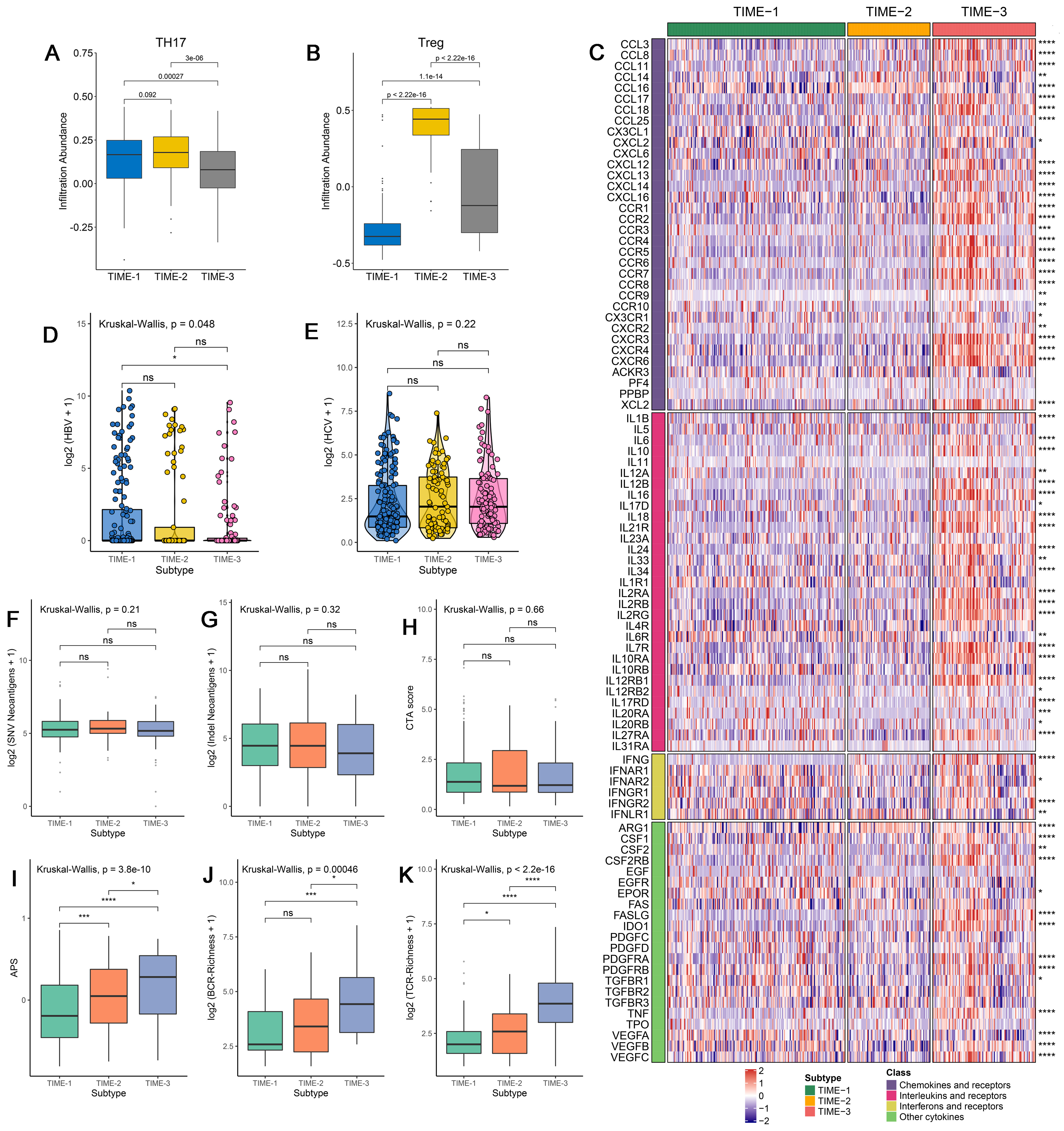

Supplement: Supplementary file 6 — Additional file 6: Fig. S4. Potential immune escape mechanisms of each phenotype. A, B, The difference of TH17 (A) and Treg (B) among the three TIME phenotypes. C, The mRNA expression of chemokines, interleukins, interferons, and other important cytokines and their receptors for each TIME phenotype. The distribution of HBV read counts (D), HCV read counts (E), SNV neoantigens (F), indel neoantigens (G), CTA score (H), APS value (I), BCR-Richness diversity (J) and TCR-Richness diversity (K) in three TIME phenotypes. For the boxplot, the asterisks represented the statistical p value (nsP > 0.05, *P < 0.05, **P < 0.01, *** P < 0.001, **** P < 0.0001). For the heatmap, the asterisks represented the statistical p value (*P < 0.05, **P < 0.01, *** P < 0.001, **** P < 0.0001). [file 12967_2020_2697_MOESM6_ESM.jpg]

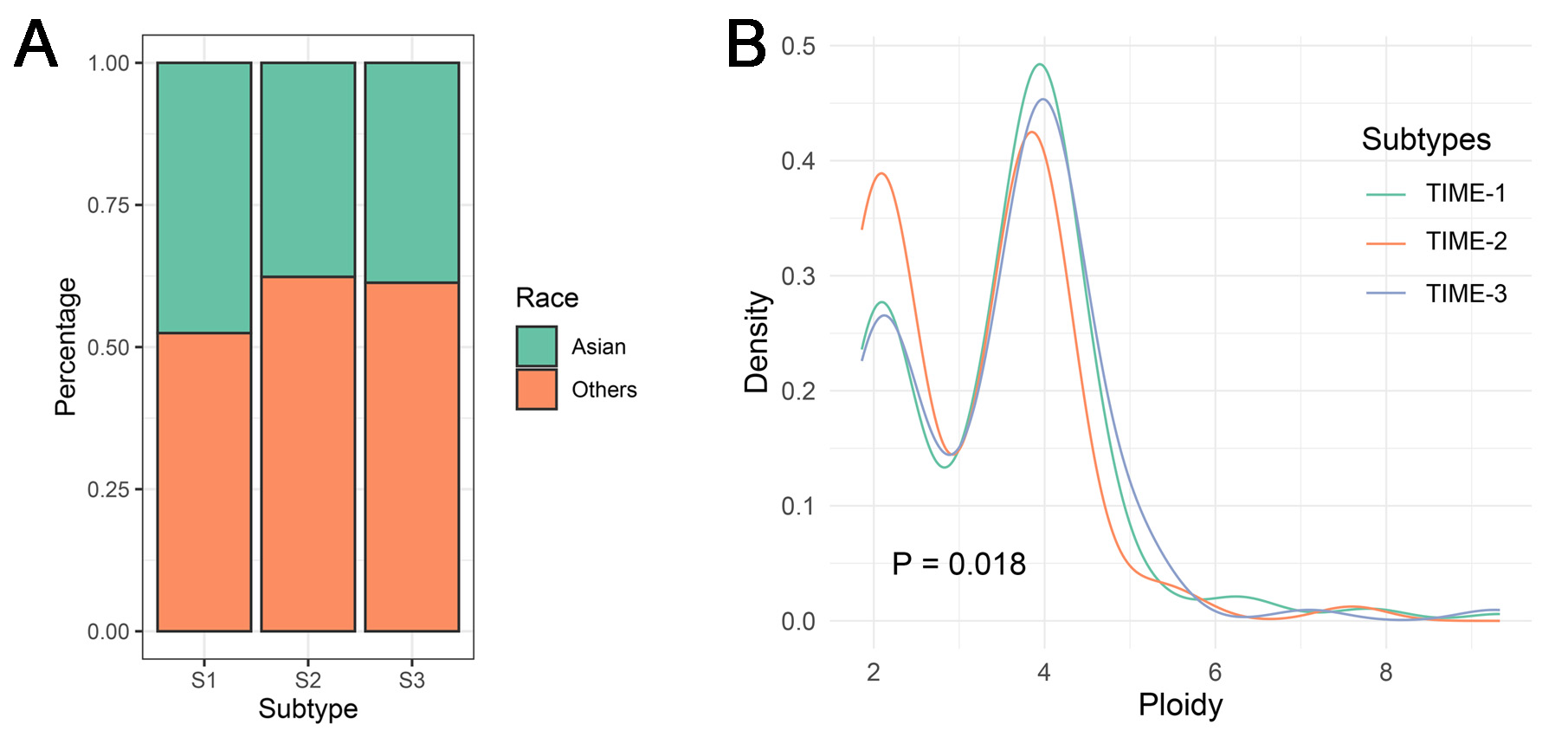

Supplement: Supplementary file 7 — Additional file 7: Fig. S5. The distribution of race and ploidy. A, The distribution of race in the TCGA cohort. B, The distribution of tumor ploidy in three TIME phenotypes. [file 12967_2020_2697_MOESM7_ESM.jpg]

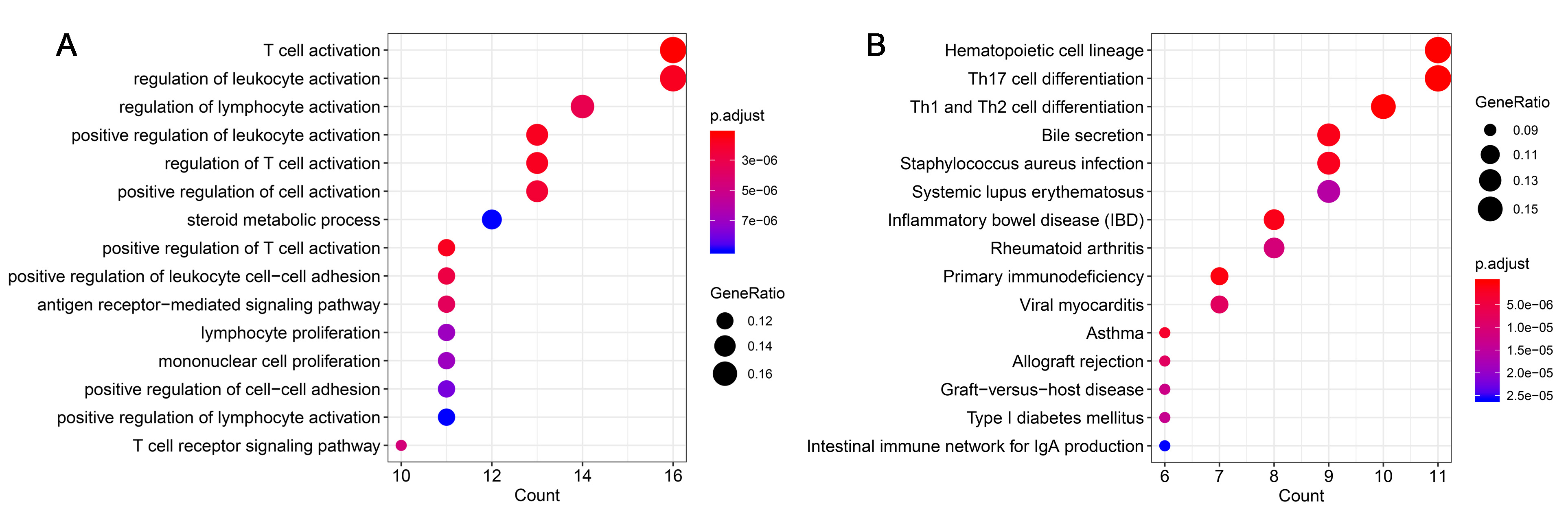

Supplement: Supplementary file 8 — Additional file 8: Fig. S6. GO and KEGG enrichment analysis of differentially expressed genes (DEGs). [file 12967_2020_2697_MOESM8_ESM.jpg]
