# Supplementary material for: A novel immune classification reveals distinct immune escape mechanism and genomic alterations: implications for immunotherapy in hepatocellular carcinoma
Source: J Transl Med. 2021 Jan 6;19:5. doi: 10.1186/s12967-020-02697-y (PMC7789239; doi:10.1186/s12967-020-02697-y)
Supplement: Supplementary file 3 — Additional file 3. Materials and methods. [file 12967_2020_2697_MOESM3_ESM.docx]

**Additiona Materials and Methods**

**Data collecting and progressing**

For the discovery cohorts, the HCC microarray datasets were recruited from Gene Expression Ominibus (GEO) database (<https://www.ncbi.nlm.nih.gov/geo/>) with the following criteria: 1) only from Affymetrix platform; 2) primary liver cancer; 3) untreated patients; 4) the number of patients ≥ 50; 5) with more than 12000 protein coding genes. Finally, fourteen eligible microarrays dataset were retrieved: GSE102079, GSE107170, GSE112790, GSE116174, GSE121248, GSE14323, NCI (National Cancer Institute) cohort (GSE14520), GSE25097, GSE45436, GSE62232, GSE63898, GSE64041, GSE76297, GSE84005 and GSE9843 (Supplementary Table S1). We processed the raw data of these datasets using Robust Multi-array Average (RMA) method implemented in affy package for background adjustment, quantile normalization and final summarization of oligonucleotides per transcript via median polish algorithm[1]. Further, the ComBat algorithm was applied to reduce the likelihood of batch effects from non-biological technical biases[2]. For the TCGA validation cohort, the TCGA-LIHC RNA-seq data was obtain from the UCSC Xena Portal (https://xenabrowser.net/datapages/), and further converted to log2 (TPM+1) which was more similar with the distribution of microarray data and more comparable between samples. Each gene expression was transformed by z-scoring across patients in both discovery and validation cohorts. Corresponding clinical and sample information were obtained from GEO and UCSC databases. For TCGA-LIHC project, the somatic mutation data, copy number alteration data, and DNA methylation data were downloaded from TCGA portal (<https://portal.gdc.cancer.gov/>). Additionally, we also download RNA-seq data and clinical information of 32 other cancer types from UCSC databases.

**Assessment of immune cells infiltration**

We performed the ssGSEA method to calculate the enrichment scores on the basis of metagenes[3]. The reason why we considered the metagene a robust approach was the two main characteristics: (1) the use of a set of genes instead of single genes that represent one immune subpopulation, because the use of single genes as markers for immune subpopulations can be misleading as many genes are expressed in different cell types; and (2) the assessment of relative expression changes of a set of genes in relation to the expression of all other genes in a sample. Referring to the Bindea et al. study[4], we incorporate 535 metagenes that represented diverse 24 immune subpopulations: innate immune cells (dendritic cells [DCs], immature DCs [iDCs], activated DCs [aDCs], plasmacytoid DCs [pDCs], eosinophils, mast cells, macrophages, natural killer cells [NKs], NK CD56dim cells, NK CD56bright cells, and neutrophils) and adaptive immune cells (B cells, T cells, T helper cells, T gamma delta [Tgd] cells , T helper 1 [Th1] cells, Th2 cells, Th17 cells, regulatory T [Treg] cells, CD8+ T cells, T central memory [Tcm] cells, T effector memory [Tem] cells, T follicular helper [Tfh] cells, and cytotoxic cells). Besides, in order to ensure the rationality and robustness of ssGSEA results, we applied another two different algorithms to further validate. The first one was CIBERSORT, a deconvolution algorithm that took a set of reference gene expression values as a minimum representation of each cell type, and based on these values, used support vector regression (SVR) to estimate the proportion of 22 immune cell types[5]. The other was the Microenvironment Cell Populations-counter (MCP-counter) method, which estimated for population of 8 immune cell by contemplating the variations of the expression degree of one gene in a specific cell type and retained the genes showing the lowest variation within the cell type[6].

**Identification and validation of the TIME phenotypes**

We used the ConsensusClusterPlus R package to determine the optimum number of clusters in GEO cohort[7]. Subsample 80% of samples at each iteration and partition each subsample into up to k (max K = 9) groups by k-means algorithm upon Euclidean distance. This process was repeated for 1,000 repetitions. Sequentially, we selected a perfect clustering result by considering the relative change in area under the cumulative distribution function curve, which was assessed by the proportion of ambiguous clustering (PAC) score, a low value of PAC implies a flat middle segment, allowing conjecture of the optimal K by the lowest PAC[8]. To further evaluate the reproducibility of the clusters generated from consensus clustering in the GEO cohort, the in-group proportion (IGP) statistical analysis was employed to demonstrate the existence of these clusters in the validation cohort (TCGA cohort). IGP was defined as the proportion of samples in a group whose nearest neighbors were also in the same group[9]. We firstly calculated a centroid for each cluster found in the GEO cohort, every sample in the TCGA cohort was classified to a cluster whose centroid had the highest Pearson correlation with the sample. Later, we implemented “clusterRepro” R package to perform IGP statistical analysis, and the statistical significance of IGP was assessed with 1000 permutations[9]. The P-value, a fraction of the null distribution IGPs more than the actual IGP of the cluster, was used to estimate cluster quality. IGP will be closed to 100% if the clusters between two cohorts are similar enough and will be closed to 0% conversely.

**Collection and analysis of tumor immunogenicity indexes**

We incorporated 14 immune-related genomic features. Tumor mutation burden (TMB) was measured as the number of coding, somatic, base substitution, and indel mutations per megabase of the targeted territory[10]. SNV or Indel neoantigens were identified through NetMHCpan v3.0[11], based on HLA types obtained from RNA-seq using OptiType (version 1.2) [12]. Aneuploidy scores (AS) were the sum of amplified or deleted (collectively ‘‘altered’’) chromosome arms[13]. Homologous recombination defects (HRD) score was determined by three separate DNA-based measures of genomic instability: large (> 15 Mb) non-arm-level regions with loss of heterozygosity (LOH), telomeric allelic imbalance (TAI), and large-scale state transitions (LST) with breaks between adjacent segments of > 10 Mb[14]. Microsatellite instability (MSI) was detect by using MSI-calling tool, MANTIS (version 1.0.3)[15]. TCR diversity (Shannon Entropy and Richness) scores were identified using MiTCR v1.0.3[16] with previously described parameters[17]. The immunoglobulin heavy chain (lgH) diversity scores (Shannon Entropy and Richness) were quantified by RSEM version 1.2.21[18], based on lgH reconstructed via the VDJer tool[19]. For both viruses (HBV and HCV), the scores of normalized reads per million (NRPM) were defined as 106 times the number of hits over the total reads in the sample. Cancer/testis-antigens (CTAs) were also involved. Antigen processing and presenting machinery (APM) scores (APS) were generated by GSVA on the basis of 18 genes relevant to APM[20]. We included the following MHC related molecules: B2M, HLA-A, HLA-B, HLA-C, HLA-DMA, HLA-DMB, HLA-DOA, HLA-DOB, HLA-DPA1, HLA-DPB1, HLA-DQA1, HLA-DQA2, HLA-DQB1, HLA-DRA, HLA-DRB1, HLA-E, HLA-F, HLA-G, TAP1, TAP2 and TAPBP. Cytolytic activity (CYT) was assessed by the geometric mean of granzyme A (GZMA) and perforin (PRF1) expression levels[21]

**Collection of cohorts with immunotherapy**

We included 6 melanoma cohorts according to the screening standard: a) the number of samples ≥ 15; b) patients with corresponding gene expression data and prognosis information of immunotherapy. Gide et al. analyzed 41 melanoma samples treated with anti-PD-1 antibody and 32 patients treated with both anti-PD-1 and anti-CTLA4 antibody [22]. The Riaz melanoma dataset consisted of 25 melanoma patients treated with anti-PD-1 antibody[23]. The Liu melanoma dataset consisted of 74 patients treated with anti-PD-1 antibody[24]. The Nathanson melanoma dataset consisted of 15 patients with anti-CTLA4 antibody [25]. In the Lauss melanoma dataset, 25 patients received treatment with anti-ACT antibody[26].

**Immunotherapy biomarkers**

To compare the accuracy of TIME index and other biomarkers in predicting immunotherapy response, we recruited 11 other biomarkers as the following description:

1. CD274, PDCD1 and CTLA4 are the immunotherapy targets approved by FDA[27]. In this study, we take the above three biomarkers as the independent variables to perform the ROC validation.
2. CD8 was estimated by the gene expression level of CD8A + CD8B;
3. TMB means the total count of non-synonymous mutations; 7 melanoma cohorts all provided mutation profile.
4. The beta chain clonality of T cell receptor CDR3 sequence was obtained using the MiXCR algorithm[28]; The immunoglobulin heavy chain clonality of B cell receptor sequence was assembled from RNA-Seq reads [28]. The predicted values of T cell clonality and B cell clonality were both calculated by $1-\sum_{i=1}^{N} p_{i} \frac{log\frac{1}{p_{i}}}{\mathrm{logN}}$ ($p_{i}$: the frequency of each receptor sequence);
5. The TIDE prediction value is computed following the procedure in the original publication [29];
6. Microsatellite instability (MSI) was predicted by ridge regression models on the basis of gene expression profile from STAD to evaluate MSI status on the website;
7. APS was quantified by the enrichment degree of the 18 genes relevant to antigen processing and presenting machinery[20];
8. CYT was assessed by the geometric mean of granzyme A and perforin expression transcript levels[21].

Subsequently, we employed the area under the ROC curve (AUC) to measure the prediction accuracy of response to immunotherapy.

All statistical analyses were performed in R version 3.6.3.

1. Irizarry RA, Hobbs B, Collin F, Beazer-Barclay YD, Antonellis KJ, Scherf U, et al. Exploration, normalization, and summaries of high density oligonucleotide array probe level data. Biostatistics. 2003; 4: 249-64.

2. Leek JT, Johnson WE, Parker HS, Jaffe AE, Storey JD. The sva package for removing batch effects and other unwanted variation in high-throughput experiments. Bioinformatics. 2012; 28: 882-3.

3. Barbie DA, Tamayo P, Boehm JS, Kim SY, Moody SE, Dunn IF, et al. Systematic RNA interference reveals that oncogenic KRAS-driven cancers require TBK1. Nature. 2009; 462: 108-12.

4. Bindea G, Mlecnik B, Tosolini M, Kirilovsky A, Waldner M, Obenauf AC, et al. Spatiotemporal dynamics of intratumoral immune cells reveal the immune landscape in human cancer. Immunity. 2013; 39: 782-95.

5. Newman AM, Liu CL, Green MR, Gentles AJ, Feng W, Xu Y, et al. Robust enumeration of cell subsets from tissue expression profiles. Nat Methods. 2015; 12: 453-7.

6. Becht E, Giraldo NA, Lacroix L, Buttard B, Elarouci N, Petitprez F, et al. Estimating the population abundance of tissue-infiltrating immune and stromal cell populations using gene expression. Genome Biol. 2016; 17: 218-.

7. Wilkerson MD, Hayes DN. ConsensusClusterPlus: a class discovery tool with confidence assessments and item tracking. Bioinformatics. 2010; 26: 1572-3.

8. Șenbabaoğlu Y, Michailidis G, Li JZ. Critical limitations of consensus clustering in class discovery. Sci Rep-Uk. 2014; 4: 6207-.

9. Kapp AV, Tibshirani R. Are clusters found in one dataset present in another dataset? Biostatistics. 2007; 8: 9-31.

10. Chalmers ZR, Connelly CF, Fabrizio D, Gay L, Ali SM, Ennis R, et al. Analysis of 100,000 human cancer genomes reveals the landscape of tumor mutational burden. Genome Med. 2017; 9: 34.

11. Nielsen M, Andreatta M. NetMHCpan-3.0; improved prediction of binding to MHC class I molecules integrating information from multiple receptor and peptide length datasets. Genome medicine. 2016; 8: 33.

12. Szolek A, Schubert B, Mohr C, Sturm M, Feldhahn M, Kohlbacher O. OptiType: precision HLA typing from next-generation sequencing data. Bioinformatics. 2014; 30: 3310-6.

13. Taylor AM, Shih J, Ha G, Gao GF, Zhang X, Berger AC, et al. Genomic and functional approaches to understanding cancer aneuploidy. Cancer cell. 2018; 33: 676-89. e3.

14. Thorsson V, Gibbs DL, Brown SD, Wolf D, Bortone DS, Yang T-HO, et al. The immune landscape of cancer. Immunity. 2018; 48: 812-30. e14.

15. Bonneville R, Krook MA, Kautto EA, Miya J, Wing MR, Chen H-Z, et al. Landscape of microsatellite instability across 39 cancer types. JCO precision oncology. 2017; 1: 1-15.

16. Bolotin DA, Shugay M, Mamedov IZ, Putintseva EV, Turchaninova MA, Zvyagin IV, et al. MiTCR: software for T-cell receptor sequencing data analysis. Nat Methods. 2013; 10: 813-4.

17. Brown PD, Patel PR. Nanomedicine: a pharma perspective. Wiley Interdisciplinary Reviews: Nanomedicine and Nanobiotechnology. 2015; 7: 125-30.

18. Mose LE, Selitsky SR, Bixby LM, Marron DL, Iglesia MD, Serody JS, et al. Assembly-based inference of B-cell receptor repertoires from short read RNA sequencing data with V’DJer. Bioinformatics. 2016; 32: 3729-34.

19. Li B, Dewey CN. RSEM: accurate transcript quantification from RNA-Seq data with or without a reference genome. BMC Bioinformatics. 2011; 12: 323.

20. Wang S, He Z, Wang X, Li H, Liu XS. Antigen presentation and tumor immunogenicity in cancer immunotherapy response prediction. Elife. 2019; 8.

21. Rooney MS, Shukla SA, Wu CJ, Getz G, Hacohen N. Molecular and genetic properties of tumors associated with local immune cytolytic activity. Cell. 2015; 160: 48-61.

22. Gide TN, Quek C, Menzies AM, Tasker AT, Shang P, Holst J, et al. Distinct Immune Cell Populations Define Response to Anti-PD-1 Monotherapy and Anti-PD-1/Anti-CTLA-4 Combined Therapy. Cancer Cell. 2019; 35: 238-55.e6.

23. Riaz N, Havel JJ, Makarov V, Desrichard A, Urba WJ, Sims JS, et al. Tumor and Microenvironment Evolution during Immunotherapy with Nivolumab. Cell. 2017; 171: 934-49.e16.

24. Liu D, Schilling B, Liu D, Sucker A, Livingstone E, Jerby-Arnon L, et al. Integrative molecular and clinical modeling of clinical outcomes to PD1 blockade in patients with metastatic melanoma. Nature Medicine. 2019; 25: 1916-27.

25. Nathanson T, Ahuja A, Rubinsteyn A, Aksoy BA, Hellmann MD, Miao D, et al. Somatic Mutations and Neoepitope Homology in Melanomas Treated with CTLA-4 Blockade. Cancer immunology research. 2017; 5: 84-91.

26. Lauss M, Donia M, Harbst K, Andersen R, Mitra S, Rosengren F, et al. Mutational and putative neoantigen load predict clinical benefit of adoptive T cell therapy in melanoma. Nat Commun. 2017; 8: 1738.

27. Nishino M, Ramaiya NH, Hatabu H, Hodi FS. Monitoring immune-checkpoint blockade: response evaluation and biomarker development. Nature reviews Clinical oncology. 2017; 14: 655-68.

28. Bolotin DA, Poslavsky S, Davydov AN, Frenkel FE, Fanchi L, Zolotareva OI, et al. Antigen receptor repertoire profiling from RNA-seq data. Nature biotechnology. 2017; 35: 908-11.

29. Jiang P, Gu S, Pan D, Fu J, Sahu A, Hu X, et al. Signatures of T cell dysfunction and exclusion predict cancer immunotherapy response. Nat Med. 2018; 24: 1550-8.
